# Electric field control of chirality

**DOI:** 10.1126/sciadv.abj8030

**Published:** 2022-01-05

**Authors:** Piush Behera, Molly A. May, Fernando Gómez-Ortiz, Sandhya Susarla, Sujit Das, Christopher T. Nelson, Lucas Caretta, Shang-Lin Hsu, Margaret R. McCarter, Benjamin H. Savitzky, Edward S. Barnard, Archana Raja, Zijian Hong, Pablo García-Fernandez, Stephen W. Lovesey, Gerrit van der Laan, Peter Ercius, Colin Ophus, Lane W. Martin, Javier Junquera, Markus B. Raschke, Ramamoorthy Ramesh

**Affiliations:** 1 Department of Materials Science and Engineering, University of California, Berkeley, Berkeley, CA 94720, USA.; 2 Materials Sciences Division, Lawrence Berkeley National Laboratory, Berkeley, CA 94720, USA.; 3 Department of Physics, Department of Chemistry and JILA, University of Colorado, Boulder, CO 80309, USA.; 4 Departmento de Ciencias de la Tierra y Física de la Materia Condensada, Universidad de Cantabria, Cantabria Campus Internacional, 39005 Santander, Spain.; 5 Center for Nanophase Materials Sciences, Oak Ridge National Laboratory, Oak Ridge, TN 37831, USA.; 6 Department of Physics, University of California, Berkeley, Berkeley, CA 94720, USA.; 7 National Center for Electron Microscopy, Lawrence Berkeley National Laboratory, Berkeley, CA 94720, USA.; 8 The Molecular Foundry, Lawrence Berkeley National Laboratory, Berkeley, CA 94720, USA.; 9 School of Materials Science and Engineering, Zhejiang University, Hangzhou, Zhejiang 310027, China.; 10 Diamond Light Source, Harwell Science and Innovation Campus, Didcot, Oxfordshire OX11 0DE, UK.

## Abstract

Polar textures have attracted substantial attention in recent years as a promising analog to spin-based textures in ferromagnets. Here, using optical second-harmonic generation–based circular dichroism, we demonstrate deterministic and reversible control of chirality over mesoscale regions in ferroelectric vortices using an applied electric field. The microscopic origins of the chirality, the pathway during the switching, and the mechanism for electric field control are described theoretically via phase-field modeling and second-principles simulations, and experimentally by examination of the microscopic response of the vortices under an applied field. The emergence of chirality from the combination of nonchiral materials and subsequent control of the handedness with an electric field has far-reaching implications for new electronics based on chirality as a field-controllable order parameter.

## Teaser

Nonlinear optics reveal how the chirality of ferroelectric vortices can be deterministically controlled using an electric field.

## INTRODUCTION

Of all the fundamental physical phenomena enabled by broken symmetry [magnetism through broken time reversal symmetry, piezoelectricity through broken spatial inversion symmetry, and the combination of these two through a toroidal order (*1*, *2*)], chirality is perhaps the most exotic and yet pervasive. It can be defined as an asymmetric configurational property where an object cannot be superimposed on its mirror image, thereby imparting a handedness. Chirality is a basic feature that determines many important properties in nature, from the strength of the weak interactions according to the electroweak theory to its essential role in the spontaneous symmetry breaking in subatomic particle physics or biophysics (*3*, *4*). A prime example of this are the building blocks of life itself, which are built up from molecules that are exclusively l-amino acids and can only convert d-glucose as a fundamental source of energy. Enantiomeric conversion of sugars such as d-glucose to l-glucose is typically accomplished by chemical means and is inherently a destructive process (the molecule is broken and reformed anew) (*4*, *5*). Chirality appears in inorganic systems, for example, in the form of spin textures in ferromagnets with broken inversion symmetry or mesoscale arrangements of molecular building blocks (e.g., liquid crystals) (*6*, *7*). Furthermore, handed responses to optical stimuli can be designed into engineered structures, such as the observation of the photonic spin Hall effect in designer metamaterials (*8*, *9*). Because of the universal nature of chirality, manipulation of the handedness by thermodynamic fields other than chemical fields would be of great interest, both scientifically and technologically with the potential for chiral electronics (*10*).

Over the past several years, nontrivial topological dipolar textures have emerged as an area of great attention in condensed matter research (*11*–*13*), building from and complementing the ongoing revolution in topological spin-based textures such as chiral skyrmions and vortices (*14*–*17*). These dipolar textures have been mainly identified in epitaxial heterostructures in which exact electrostatic and elastic boundary conditions are imposed on a crystalline ferroelectric layer, such as PbTiO_3_ (*13*, *18*, *19*). Dipolar textures in ferroelectrics, such as polar vortices and skyrmions, have been extensively studied, and several interesting physical phenomena have been found, such as toroidal order and negative capacitance (*20*–*22*). Perhaps the most exotic and unexpected among them is the emergence of chirality (i.e., handedness) in polar vortices and skyrmions that arise from a sequence of achiral materials (*20*, *23*). The existence of chiral behavior in these systems was demonstrated using resonant soft x-ray diffraction–based circular dichroism (RSXD-CD) measurements (*20*, *24*, *25*). However, the use of these synchrotron-based approaches, while scientifically sophisticated, can limit the pervasive impact of these chiral textures, particularly for possible applications in next-generation technologies. In parallel, strain engineering or electric field manipulation and control of chiral phases would be of fundamental and technological interest. This has been the subject of theoretical studies (*26*–*28*) but has remained elusive from the experimental point of view. A recent work addresses this problem, but the presence of random fields during the switching precludes a deterministic control of the vortex rotation reversal (*29*). With this as background, the present work introduces two main innovations: (i) the manipulation of the chirality in a controlled, deterministic, and reversible way in polar vortices over mesoscale regions using an electric field and (ii) the use of conventional optical techniques to probe chirality in these polar textures.

## RESULTS AND DISCUSSION

**Fig. 1.**
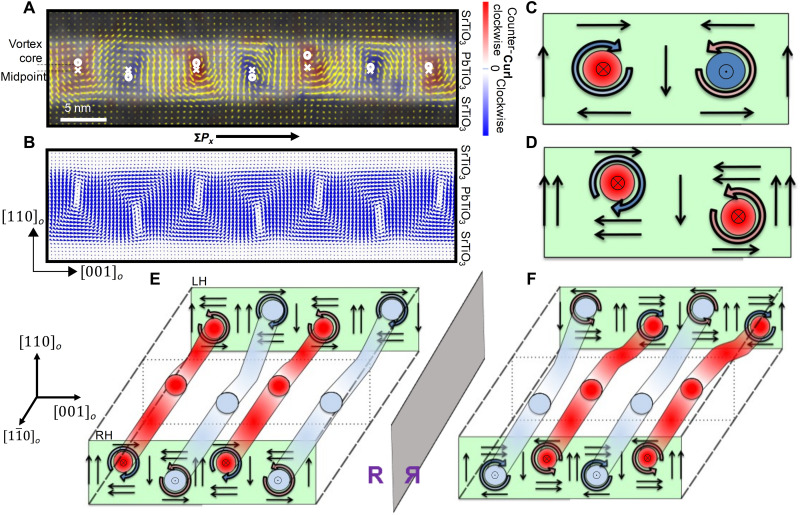
Different mechanisms for achieving chirality. (**A**) HAADF-STEM displacement map of polar vortex array. Colors indicate the curl of the local polarization. Circles represent the core of the vortices, and crosses represent the center points of the PbTiO_3_ layer. (**B**) Phase-field modeling of vortex array demonstrating pronounced buckling of vortices. (**C**) CW (blue curled arrow) and CCW (orange curled arrow) vortices coupled with antiparallel axial components of the polarization. Filled red and blue circles represent the direction of the axial polarization (along the ${\left[1\bar{1}0\right]}_{o}$ direction), and light colors represent lower values. Straight arrows represent the direction of the local polarization along the [001]*_o_* (horizontal) or [110]*_o_* (vertical) directions. Within the right-hand rule discussed in (*20*), both vortices in the sketch are right handed. (**D**) Asymmetry between up- and down-polarized domains (along [110]*_o_*), superimposed on an offset that generates a mismatch between left and right polarization along the [001]*_o_* direction. In the sketch, at the center of the ferroelectric layer, the two vortices point predominantly to the left. The handedness of the two vortices is opposite, but one of them is slightly larger than the other, giving the whole system a net chirality. (**E**) Schematic representation of the right-handed and left-handed domains experimentally observed, separated by a domain wall (dashed square at the center). The two sources of chirality (antiparallel axial components at the center of consecutive vortices plus an offset of its center) coexist within a domain. At the domain wall, the sense of rotation of a vortex is reversed, keeping constant the sense of the axial polarization. (**F**) Vortices of (E) after performing a mirror symmetry operation, represented by “R.” Schematics with the three orthogonal reflections of the polar texture supercell to determine their chiral nature are presented in fig. S1.

For this work, symmetric (SrTiO_3_)_20_/(PbTiO_3_)_20_/(SrTiO_3_)_20_ (STO/PTO/STO) trilayers were synthesized on orthorhombic DyScO_3_ (DSO) (110) substrates using reflection high-energy electron diffraction (RHEED)–assisted pulsed laser deposition [Materials and Methods and (*13*)]. In these heterostructures, the vortex state dominates because of the interplay between depolarization energy at the PTO/STO interfaces, elastic energy from the tensile strain imposed by the DSO substrate, and gradient energy in the ferroelectric (*13*, *20*). High-angle annular dark-field scanning transmission electron microscopy (HAADF-STEM) in conjunction with vector displacement mapping was used to visualize the local ion displacements in this vortex phase (Fig. 1A), which reveal vortices with alternating clockwise (CW)/counterclockwise (CCW) polarization curls in the PbTiO_3_ layer. The cores of these neighboring vortices, indicated by white circles, are displaced vertically in opposite directions from the midpoint (indicated by white crosses in Fig. 1A). As a consequence, this buckling (i.e., a staggered vortex configuration) leads to a net in-plane polarization along the [001]*_o_* direction, which is validated by phase-field modeling (Fig. 1B; details of the phase-field modeling are provided in Materials and Methods).

Chirality can arise in ensembles of these polar vortices as a consequence of a few symmetry-breaking pathways, which are schematically illustrated (Fig. 1, C to E). As has been previously shown (*20*), chiral behavior can arise because of the coexistence of the axial component of polarization (perpendicular to the plane defined by the vortices; here, the ${\left[1\bar{1}0\right]}_{o}$ direction) with the vorticity of the CW and CCW vortices (*23*). Mathematically, it is described by the helicity ℋ, defined as

$$H=\int p∙(\vec{\nabla }\times p)d^{3}r$$ (1)

where **p** is the local value of polarization, thus leading to either a left-handed (negative value of ℋ) or right-handed (positive ℋ) vortex array that is controlled by the axial polarization direction, as schematically described in Fig. 1C. How the lateral and axial components of the polarization are measured is described in Materials and Methods and represented below. A second source of chirality can be observed when the vortices are offset along the [110]*_o_* direction, normal to the vortex axis (Fig. 1D). These misalignments lead to a mismatch in the polarization pointing along the [001]*_o_* direction (left/right). If this offset appears in combination with a nonequal fraction of dipoles pointing along the vertical [110]*_o_* direction (i.e., if the up and down domains do not exactly equally match in size), then an excess/deficit of the CW/CCW rotations is generated, making the whole system chiral (see fig. S2). Therefore, for given sizes of the up and down domains, the structure exhibits a chirality that is dependent on the direction of the mismatch (or buckling). From a quantitative point of view, the degree of chirality can be captured by the helicity defined earlier. While the sign of the helicity allows an unambiguous classification of the handedness of a given array of polar vortices, its absolute magnitude determines the strength of the chiral behavior (e.g., the magnitude of CD; see section S4 for details of the strength of the chirality). The second pathway (Fig. 1D) produces helicities that are smaller in magnitude than those of the first (Fig. 1C) and arises from an imbalance in the center of rotation of the two vortices. This pathway has the advantage, however, that it is potentially easier to deterministically reverse the vortex offset with an external electric field applied perpendicular to the lateral component of the polarization (fig. S1B) and thus the net left/right polarization direction. The different paths for switching the chirality of the model depicted in Fig. 1C are energetically costly or impractical (fig. S3). Experimentally, we observe vortex structures that have attributes of both sources of chirality (Fig. 1, C and D), i.e., an antiparallel axial component that is superimposed on an up/down shift of the CW/CCW rotating vortices. Experimentally, domains with different chirality separated by a thin domain wall are observed, as schematically shown in Fig. 1E. At the domain wall, the rotation of a given tube is reversed, with a corresponding change in the position of the vortex core, keeping constant the axial component of the polarization. This domain structure is chiral because it cannot be superimposed to any mirror image (Fig. 1F and fig. S1C). Moreover, reversal of the net polarization along the [001]*_o_* direction results in a reversal of the buckling pattern, providing a possible pathway to switch the chirality with an electric field. We demonstrate such an electric field–driven reversal of the chirality using optical second-harmonic generation–based CD (SHG-CD) as the indicator of chirality (Materials and Methods) and validated by electric field–dependent electron microscopy studies.

The SHG-CD studies were carried out using a confocal microscope with a spatial resolution of ~300 nm as a convenient, laboratory-based approach to study chirality (Materials and Methods and section S5). SHG is a photonic exchange between the frequency components of the electromagnetic field, during which two photons of lower-frequency ω are absorbed and one photon of 2ω is created in a single quantum-mechanical process. SHG fundamentally arises from symmetry breaking in the second-order susceptibility, making it a powerful tool for imaging crystal lattices with symmetry breaking, such as ferroelectrics (*30*). Moreover, CD in SHG is an established technique to probe chirality (*31*–*34*) and can result from, for instance, chiral ordering of electric dipoles or the existence of ferromagnetism (*35*, *36*). The lack of magnetism in PTO and STO suggests that any SHG-CD that arises must come from a chiral ordering of dipoles. Such a natural CD (NCD) of nonmagnetic origin can only arise from the parity-odd event of three electric dipole transitions. Although all noncentrosymmetric materials can display such a transition, only when helical light interacts with a handed environment will the magnitude of the NCD be nonzero (details of the full derivation are provided in section S7) (*35*).

**Fig. 2.**
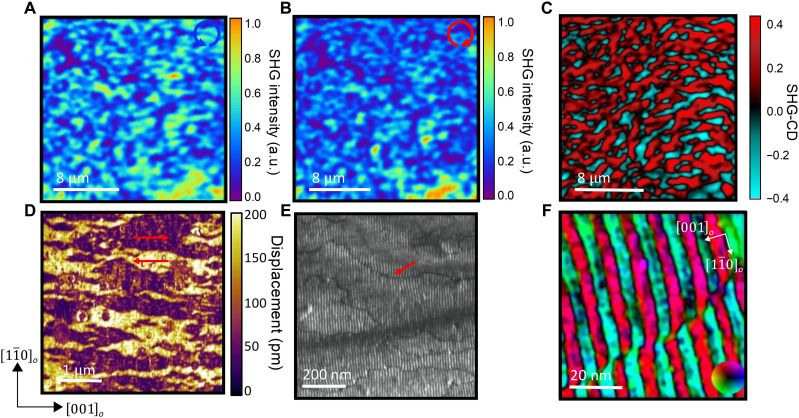
Vortex domain structure. SHG images taken with (**A**) right circularly (RC) and (**B**) left circularly (LC) polarized excitation. (**C**) SHG-CD calculated from the images shown in (A) and (B) using Eq. 2. (**D**) Lateral PFM image, with red arrows indicating regions of opposite polarization along [001]*_o_*. (**E**) Weak-beam dark-field TEM image (DF-TEM) image of vortex domains. The arrow shows the location of antiphase domain wall. (**F**) HAADF-STEM displacement map at a vortex domain wall. Colors indicate the direction of atomic displacement with respect to the color wheel. a.u., arbitrary units.

Confocal scanning microscopy images of the SHG signal with right (RC) and left (LC) circularly polarized excitation are shown (Fig. 2, A and B, respectively). The SHG-CD signal is then calculated from the asymmetry of intensity distribution between the two images

$$\frac{I_{\text{LC}}-I_{\text{RC}}}{I_{\text{LC}}+I_{\text{RC}}}$$ (2)

where *I*_LC_ and *I*_RC_ are the SHG intensities when excited by an LC and RC polarized beam, respectively. The resulting CD image (Fig. 2C) reveals regions with strong SHG-CD signals (magnitude as large as 0.4), strongly pointing to the existence of chiral ordering. Notably, domains with opposite CD elongated along the [001]*_o_* direction are visible, indicating domains or regions of vortices with opposite chirality. Note that while the characteristic size of the domains imaged in SHG-CD is ~1 μm, this is close to the diffraction-limited spatial resolution of the microscope (~300 nm with an oil immersion lens) and does not rule out the existence of features below this length scale.

In-plane piezoforce microscopy (PFM) studies reveal a lenticular-shaped domain pattern that is captured in Fig. 2D, with antiparallel in-plane polarization components identified by the red arrows. The lateral length scales of these domains are in the 100- to 300-nm range, while they extend to ~1 μm in the longitudinal direction ([001]*_o_*). The ferroelectric domains are qualitatively similar in morphology to the chiral domains in the SHG-CD image and suggest a possible causal dependency between them. At higher resolution, for example, in a weak-beam dark-field TEM image (DF-TEM) (Fig. 2E), we are able to observe the arrays of vortices with their long axis along the ${\left[1\bar{1}0\right]}_{o}$ direction (vertically in Fig. 2E). We observe contrast arising from antiphase domain walls, indicated by the red arrow. At higher resolution, for instance, using HAADF-STEM–based atomic imaging, we can discern the displacement maps of the atomic structure across these interfaces, as illustrated in Fig. 2F.

**Fig. 3.**
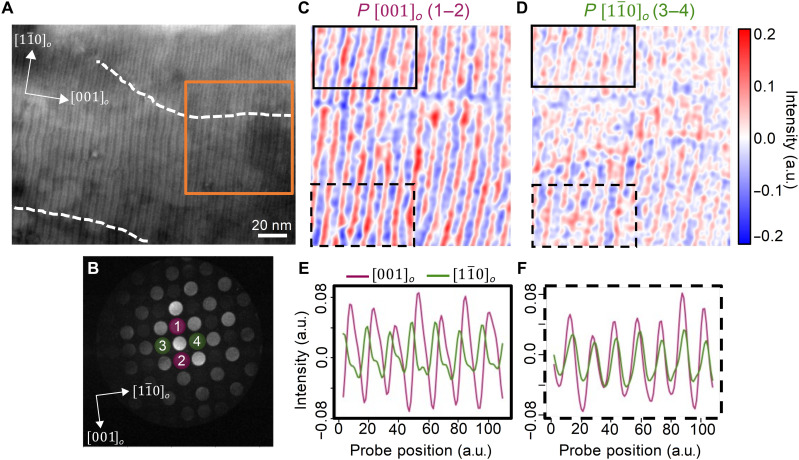
4D STEM analysis of in-plane vortices. (**A**) Large-area HAADF-STEM showing the in-plane vortices along the ${\left[1\bar{1}0\right]}_{o}$ direction with domain boundaries indicated by the gray dotted line. (**B**) A representative CBED pattern from the vortex structure is shown in (A), which identifies the reflections that were used to form the 4D STEM images in (C) and (D). (**C**) Lateral and (**D**) axial polarization components extracted from orange square region indicated in (A). (**E** and **F**) Line scans from different regions across the domains, which show lateral and axial polarization out of phase and in phase, respectively.

We next focus on the relationship between the lateral and axial components of the polar structural distortion across these boundaries. Because HAADF-STEM is mainly a two-dimensional (2D) projection technique, it cannot deterministically probe the axial component. Instead, we used 4D STEM to probe the in-plane and axial polarization components. In 4D STEM, a focused probe is scanned across the sample region to obtain a convergent beam electron diffraction (CBED) pattern at every scan position. Unlike HAADF-STEM, 4D STEM has depth dependence, providing additional information through the thickness of the sample (*37*, *38*). This technique was used by Nguyen *et al.* (*39*) to identify the in-plane and axial polarization in vortices. All the analysis of our 4D STEM images was carried out using the py4DSTEM software package (described in section S8) (*40*). Because of this advantage, it is easier to measure and distinguish between the lateral and axial polarization. Figure 3A shows the HAADF image where 4D STEM was performed, and Fig. 3B shows the CBED pattern averaged over the entire image in Fig. 3A. To study the change in chirality across a domain boundary (white dashed line in Fig. 3A), we obtained polarization maps by subtracting the normalized intensity of the virtual images formed from the Friedel pair CBED disks along the lateral (disk 1 and disk 2) and axial (disk 3 and disk 4) directions (see fig. S6). Polarization maps of the lateral and axial component (Fig. 3, C and D, respectively) were generated from within the orange boxed region in Fig. 3A. Further details of this 4D STEM imaging are described in Materials and Methods and section S8. From these images, we obtain the line scans from the two regions across the domain boundary (Fig. 3, E and F). Focusing on the region outlined by the solid black line in Fig. 3 (C and D), we see that the lateral and axial components are in phase with one another, as shown in Fig. 3E. In contrast, in the region outlined by the dashed black lines, the lateral and axial components are out of phase with each other. Such a change in the phase in the lateral and axial components has been shown to be an indicator of the change in the chirality (*39*).

These results provide the ingredients that support the chiral and switchable atomic model schematized in Fig. 1E, in particular, (i) the observation of alternating CW and CCW vortices running along the ${\left[1\bar{1}0\right]}_{o}$ direction, visualized in the red and blue stripes for the [001]*_o_* component of the polarization in Fig. 3C; (ii) the existence of antiparallel axial components of the polarization along the ${\left[1\bar{1}0\right]}_{o}$ axial direction in consecutive vortices, as shown also by the red and blue stripes in Fig. 3D; (iii) the pronounced buckling of the vortices observed by HAADF-STEM in Fig. 1A; and (iv) the presence of domains of different helicity, separated by the domain wall marked with the dotted lines in Fig. 3 (A, C, and D). The character of the domains is clearly visible in Fig. 3 (E and F), where the components of the polarization along the [001]*_o_* and ${\left[1\bar{1}0\right]}_{o}$ directions are out of phase and in phase, respectively, indicating a change in the handedness, as described schematically in Fig. 1E.

**Fig. 4.**
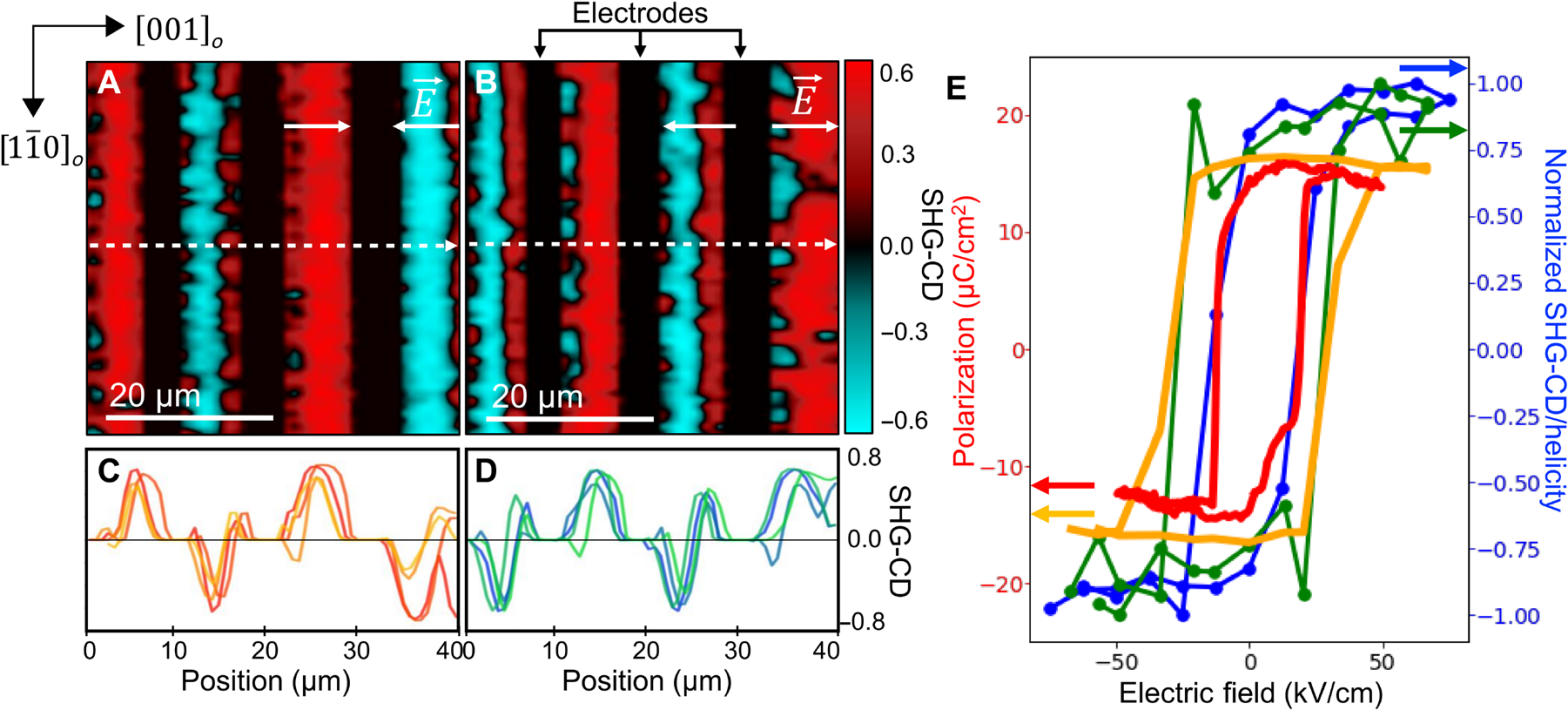
Electric field switching of chirality. (**A** and **B**) SHG-CD for alternating applied electric fields of ±50 kV/cm as indicated by the white arrows. (B) SHG-CD for the same region shown in (A) but with inverted electric field polarities. (**C** and **D**) Data along dashed white lines in (A) and (B) with repeated reversal of the electric field polarity. Subsequent scans are shown going from red/blue to orange/green for (A)/(B). (**E**) Hysteresis of the chiral switching measured using SHG-CD (blue) and calculated helicity (green), both of which are normalized. Polarization was measured from an electric field applied along the [001]*_o_* in-plane direction (red) and calculated polarization hysteresis curve from second principles (orange).

Having established the equilibrium chiral domain structure, we next turn to their in situ manipulation during the SHG-CD measurements. A DC electric field was applied along the in-plane [001]*_o_* direction using lithographically patterned interdigitated electrodes (fig. S7, A and B). This pattern leads to linear electrostatic potentials, i.e., constant electric fields of opposite polarity for adjacent interdigitated regions (as indicated by the white arrows; Fig. 4, A and B). The direction of the DC field favors the [001]*_o_* component of the polarization in the same sense and, therefore, selects the direction of the buckling of the vortices. This offset of the vortex cores determines unequivocally the helicity, as shown in Fig. 1 (E and F), and is supported by the second-principles simulations in fig. S10. Therefore, the chirality can be deterministically controlled by the external field. An SHG-CD image taken with an applied field of ±50 kV/cm (±40 V across an 8-μm electrode spacing) within neighboring interdigitated regions is shown (Fig. 4, A and B). The applied electric field leads to the formation of large bands of uniform CD across the entire region between the interdigitated electrodes, with the sign of the CD response being the measure of the sign of the chirality. As expected, reversing the sign of the applied voltage switches the sign of the CD (chirality) in each region between the electrodes. The robustness of the switching is illustrated by the line profiles (Fig. 4, C and D), which correspond to SHG-CD along the white dashed lines (Fig. 4, A and B) for a sequence of opposite polarity fields. SHG-CD in these saturated regions is also larger in magnitude (by ~50%) than that observed in the unmodified samples. This increased magnitude likely arises because of two factors. First, the average size of polar domains in the virgin sample is on the order or even smaller than the diffraction-limited imaging resolution of the SHG measurements, which leads to averaging over regions with positive and negative CD responses, consequently decreasing the magnitude of the resultant CD. Second, the application of an electric field induces larger offsets between the core of the vortices and, therefore, there is an enhancement of the helicity.

The DC voltage dependence of the SHG-CD measurements (Fig. 4E, blue data) reveals a CCW, hysteretic nature of the field dependence of the normalized SHG-CD (chirality) with a coercive field of ~20 kV/cm and a nonvolatile remanent chiral state. This is a direct indication of the switchability of chirality by an electric field. Data illustrating reproducibility of the hysteresis effect are also presented in fig. S8 (A and B). To probe this further, we carried out polarization–electric field hysteresis measurements using the same interdigitated electrodes to probe the switching of the in-plane component of the switchable polarization (Fig. 4E, red data). The raw data for this ferroelectric hysteresis loop measurement (fig. S9) show the existence of a linear dielectric component superimposed on the ferroelectric component. For the sake of simplicity, we have subtracted out the linear dielectric component (Fig. 4E). The overlap of the ferroelectric polarization hysteresis loops with the SHG-CD hysteresis establishes a direct correlation between the in-plane polarization (arising from the buckling pattern in the vortex arrays) and the consequent chiral behavior as discerned from the SHG-CD measurements.

To shed light on the microscopic origin of the switchable chirality, particularly the change in the sense of buckling of the vortices, we have carried out second-principles simulations (*41*, *42*) on (PbTiO_3_)_10_/(SrTiO_3_)_10_ superlattices, using the same lattice potential as in (*20*) (see Materials and Methods). The polarization textures obtained after the relaxation of the atomic structures are similar to those previously reported (*20*), i.e., the local dipoles display pairs of CW and CCW vortices alternating along the [001]*_o_* axis. The imposed biaxial tensile strain favors the development of a net in-plane polarization superimposed on the previous structure. As a consequence, and to accommodate the onset of this polarization, neighboring vortices are arranged in a buckled geometry. Two enantiomorphic structures are possible (fig. S10, A and B), where it is seen how the direction of the in-plane polarization unequivocally determines the offset between the core of the vortices. A polarization pointing along the positive (negative) [001]*_o_* forces the center of the CW vortex to be lower (higher) than the core of the neighboring CCW vortex (Fig. 1, E and F).

The ferroelectric hysteresis loops for the net in-plane polarization and the normalized integrated helicity are compared in Fig. 4E (orange and green), respectively. When the external electric field points in the same direction as the net in-plane polarization, the offset between the vortices increases. This tendency is maintained until the core of the two vortices touch the PTO/STO interface (fig. S10, C and D). Beyond these fields, the vortices are destroyed, transforming themselves first in sinusoidal (wave-like) textures and finally into achiral, monodomain configurations (zero helicity) with all the polarization pointing in plane. The opposite happens when the external electric field points in the opposite direction to that of the in-plane polarization. In that case, the in-plane component of the polarization is progressively reduced, with a concomitant reduction of the offset (fig. S10, E and F). Beyond the coercive field, the in-plane polarization is reversed, followed by the direction of the offsets and the integrated helicity. The theoretically calculated hysteresis in the polarization, with the linear dielectric component subtracted, and helicity of the vortices (Fig. 4E) are in close agreement with the experimental data, with the coercive field being slightly larger at ~25 kV/cm. Polarization hysteresis raw data are also provided (fig. S9).

**Fig. 5.**
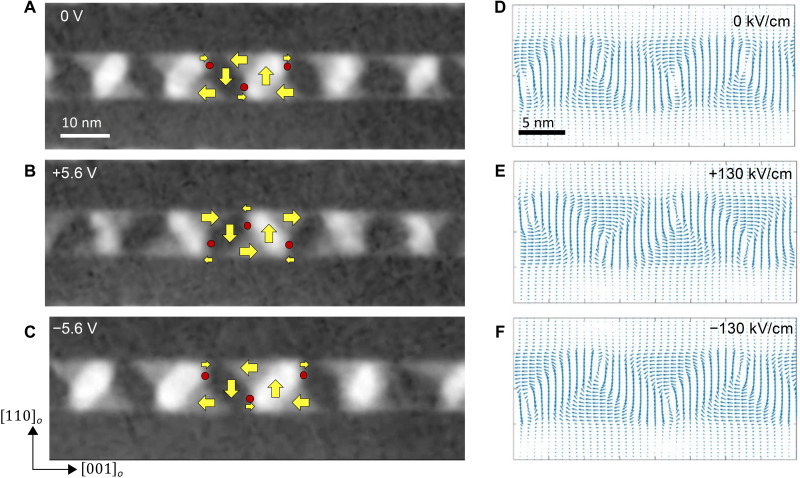
Reversal of vortex buckling. (**A** to **C**) DF-TEM showing atomic-scale restructuring during chiral phase switch. Yellow arrows indicate the direction of polarization, and red circles indicate the vortex core location. (**D** to **F**) Phase-field modeling demonstrating the reversal of buckling pattern with applied field.

We then probed the microscopic origins of this correlation between the buckling of the vortices and chirality as well as the electric field manipulation of the buckling/chirality using cross-sectional DF-TEM studies. Dark-field images were obtained using the [110]*_o_* and [001]*_o_* reflections (details of the full diffraction analyses and the dark-field images are presented in fig. S11). From the combination of the diffraction condition and the image contrast, we are able to assign vectorial directions to the intensity maps (Fig. 5A); the contrast in the DF-TEM distinguishes regions of opposite polarization along the out-of-plane [110]*_o_* direction. The arrows show the direction of the polarization within each region, and the magnitude of the arrows represents the area of each region. A characteristic chevron-shaped pattern can be seen, and the intersections of the four regions represent the core of the vortex (red dots; Fig. 5, A to C). In situ electric field studies were carried out (fig. S13) on such a vortex array. With increasing electric field, the neighboring vortices flip the sense of their in-plane component at a voltage of ~5.6 V. This is schematically described by the red circles and yellow arrows (Fig. 5, A to C). This flipping (Fig. 5B) is a reversal of the buckling pattern described theoretically in Fig. 1 (B to D) and is indicative of a change in chirality, as inferred from the model (Fig. 1, E and F). Upon reversing the voltage (Fig. 5C), the buckling pattern of the vortices is flipped back to the original state. Phase-field simulations that recreate the structures produced in the in situ TEM data (Fig. 5, D and F) illustrate the switching of the vortex buckling under the application of an electric field along the [001]*_o_* direction.

In summary, this work presents two broad implications. First, it has been shown that SHG-CD measurements using laboratory-based confocal optical measurements can be an effective probe of the chirality in these vortex arrays with a spatial resolution in the range of ~300 nm. In turn, these studies show that the vortex arrays assemble into domains of opposite chirality, identified by the change in the sign of the CD. The coexistence of regions of opposite CD (i.e., chirality) points to an equilibrium between regions of opposite chirality and therefore to a pathway by which one region can be converted into the other with a thermodynamic field. Of greater importance is the observation that an electric field can switch the sign of the local chirality in a reversible, controlled, and deterministic fashion. To the best of our knowledge, this is one of but a few instances where the chirality of a material can be manipulated with thermodynamic fields other than the chemical field. This work also points to a host of possibilities including probing the dynamics of this chiral switching, the role of the chiral-domain boundary, and the microscopic structure of these domain boundaries.

## MATERIALS AND METHODS

### Sample preparation

The [(PbTiO_3_)*_n_*/(SrTiO_3_)*_n_*] (*n* = 16 and 20) trilayers were synthesized on single-crystalline DSO (110) substrates via RHEED-assisted pulsed laser deposition (KrF laser). PbTiO_3_ and SrTiO_3_ were grown at 610°C in 100 mtorr of oxygen pressure. For all materials, the laser fluence was 1.5 J/cm^2^ with a repetition rate of 10 Hz. RHEED was used during the deposition to ensure the maintenance of a layer-by-layer growth mode for both PbTiO_3_ and SrTiO_3_. The specular RHEED spot was used to monitor the RHEED oscillations. After deposition, the trilayers were annealed for 10 min in 50 torr of oxygen pressure to promote full oxidation and then cooled down to room temperature at that oxygen pressure.

### SHG-CD measurement

SHG-CD measurements were made in the reflection mode, using a Ti/sapphire oscillator for excitation with ~100-fs pulses and center wavelength of 800 nm, a 78-MHz repetition rate, and an average power of 1 mW as shown in fig. S4. Pulses with linear polarization were sent through a one-fourth wave plate (l/4) to generate LC or RC polarized excitation, and the light was then sent through a beam splitter and focused on the sample using an oil immersion objective lens [numerical aperture (NA) = 1.4]. The back-scattered SHG signal was sent through a short-pass filter and detected using a spectrometer (SpectraPro 500i, Princeton Instruments) with a charge-coupled device camera (ProEM+: 1600 eXcelon3, Princeton Instruments). Confocal scanning microscopy was used to create images of the SHG intensity generated with LC polarized excitation light and, subsequently, with RC polarized excitation light. These were used to obtain images by calculating the CD at each image pixel.

### Capacitance measurements

The in-plane polarization versus electric field measurements were performed using interdigitated electrodes because there is no bottom electrode. The electrodes were, at first, patterned using a photolithographic lift-off process with g-line photoresist, followed by Pt sputtering (100 nm) as the electrode. The photoresist was removed by sonicating with acetone, rinsed in deionized water, and blow-dried with nitrogen. The permittivity of the film was determined from the measured capacitance using the partial capacitance model as described by Farnell *et al*. (*43*). In our case, the length of the fingers was 500 μm, the distance between two fingers was 8 μm, and the number of fingers was 40.

### TEM measurements

The planar view TEM sample was mechanically polished using Allied High Tech MultiPrep at 0.5° wedge angle, then ion-milled initially at 4 keV, and finished with 200 eV for final cleaning using a Gatan Precision ion polishing system. To image the local microstructure and detect the polarization distribution of the planar view TEM sample, atomic-resolution STEM images were acquired on a spherical aberration (Cs)–corrected FEI Titan 80-300 microscope operated at 300 kV with a point-to-point resolution of 50 pm at the National Center for Electron Microscopy, Lawrence Berkeley National Laboratory. Pairs of orthogonal scan images were used to correct the nonlinear drift distortions of the microscope in STEM mode. Using an established displacement vector-mapping algorithm (*13*, *22*), the local noncentrosymmetry of atomic columns in the ABO_3_ lattice was probed to determine the polar structures.

### 4D STEM measurements

4D STEM measurements were carried out using a Gatan K3 direct detection camera located at the end of a Gatan Continuum imaging filter on a TEAM I microscope (aberration-corrected Thermo Fisher Scientific Titan 80-300). The microscope was operated at 300 kV with a probe current of 100 pA. The probe semiangle used for the measurement was 2 mrad. Diffraction patterns were collected using a step size of 1 nm with 514 by 399 scan positions. The K3 camera was used in full-frame electron counting mode with a binning of 4 pixels by 4 pixels and camera length of 1.05 mm. The exposure time for each diffraction pattern was 47 ms.

### Phase-field simulations

In the phase-field modeling of the PbTiO_3_/SrTiO_3_ multilayer system, the evolution of the polarization was obtained by solving the time-dependent Ginzburg-Landau equations

$$\frac{\partial P_{i}(r,t)}{\partial t}=-L\frac{\delta F}{{\delta P}_{i}(r,t)}(i=1,2,3)$$ (3)

where *L*, *r*, and *t* denote the kinetic coefficient, spatial position vectors, and time, respectively. The contributions to the total free energy *F* include the Landau bulk energy, elastic energy, electric (electrostatic) energy, and gradient energy, i.e.,

$$F=\int (f_{\text{Landau}}+f_{\text{Elastic}}+f_{\text{Electric}}+f_{\text{Gradient}})\mathrm{dV}$$ (4)

Expressions for each energy density can be found in the literature (*44*). Because of the inhomogeneity of elastic constants in the [(SrTiO_3_)_9_/(PbTiO3)_9_]*_n_* multilayer system, a spectral iterative perturbation method was used to solve the mechanical equilibrium equation to obtain the stress field (*45*). The pseudocubic lattice constants for PbTiO_3_ and SrTiO_3_ were taken as 3.9547 and 3.905 Å, respectively (*45*), while the anisotropic in-plane lattice constants for the substrate DSO is taken from the literature to calculate the misfit strain (*46*). Material constants for PbTiO_3_ and SrTiO_3_ used in the simulations are found from the literature, and these include the Landau potentials, elastic constants, electrostrictive coefficients, background dielectric constants, and gradient energy coefficients (*45*). 3D phase-field simulation of the [(SrTiO_3_)_9_/(PbTiO_3_)_9_]*_n_* multilayer system is done using discrete grids of (120∆*x*) × (120∆*y*) × (350∆*z*) with ∆*x* = ∆*y* = ∆*z*= 0.4 nm, where ∆*x*, ∆*y*, and ∆*z* are in real space. The thickness of the substrate, film, and air is 15∆, (18*n* + 9)∆*z*, and (326 − 9*n*)∆*z*, respectively, where *n* is the number of the confined PbTiO_3_ layer. In the film, alternating nine grids of PbTiO_3_ layers and nine grids of SrTiO_3_ layers are incorporated to simulate the multilayer system. The periodicity of PbTiO_3_ and SrTiO_3_ layers effectively describes the experimental observation. Periodic boundary conditions are assumed in both the *x* and *y* directions, and a superposition method is used along the *z* direction (*47*). Random noise is used as the initial setup to simulate the thermal fluctuation during the annealing process.

### Second-principles calculations

The second-principles simulations were performed using the same methodology presented in previous works (*41*, *48*), as implemented in the Scale-Up package (*41*, *42*). The interatomic potentials, and the approach to simulate the interface, are the same as in (*20*). We impose an epitaxial constraint assuming in-plane lattice constants of *a* = *b* = 3.911 Å forming an angle of γ = 90°. This corresponds to a small tensile epitaxial strain of +0.25% with respect to the reference structure used in previous works (where *a* = *b* = 3.901 Å) and mimics the mechanical boundary conditions imposed by the DSO substrate. This epitaxial condition favors the onset of an in-plane component of the polarization, which couples with the offset of the cores of the vortices.

For computational feasibility, we have focused on a simulation supercell made from a periodic repetition of 2*n* × 1 × 2*n* elemental perovskite unit cells, sufficiently large to simulate domains in the *n* = 10 superlattice. At low temperatures, the vortices do not vary along the axial ${\left[1\bar{1}0\right]}_{o}$ direction. Therefore, the simplification of taking only one unit cell along this direction does not affect the model while it speeds up the calculations. For a given value of the electric field, we solved the models by running Monte Carlo–simulated annealing down to very low temperatures, typically comprising 10,000 thermalization sweeps, followed by a conjugate gradient relaxation to find the ground-state or metastable solutions. Local polarizations are computed within a linear approximation of the product of the Born effective charge tensor times the atomic displacements from the reference structure positions divided by the volume of the unit cell. From the complex polarization texture, we computed the helicity following Eq. 1. The curl of the local polarization that appears in the integrand is computed by a five-point finite difference central method. To improve the numerical stability of the derivatives, we interpolated the polarization array via cubic splines to generate a fine-grained grid.

The experimentally observed samples correspond to the structure sketched in Fig. 1E, where the antiparallel axial components of the polarization in consecutive vortices coexist with an offset of the core of the vortices. This can be considered as a juxtaposition of the models sketched in Fig. 1 (C and D). As probed in fig. S10, the switching of chirality in these structures is totally due to the switching of chirality of the model depicted in Fig. 1D. For computational feasibility, we focus on this model, saving computational resources because it does not require the presence of domains. For that, we apply, on the one hand, a constant external field along the ${\left[1\bar{1}0\right]}_{o}$ direction to force the parallel orientation of the axial [Bloch (*41*, *48*)] component of the polarization at the core of neighboring polar vortices. On the other hand, another constant external field is applied along the [110]*_o_* direction to favor one of the out-of-plane domains (the one parallel to the external field) against the other. As shown in section S1, the final structure is chiral because it cannot be mapped by any combination of rotations and/or translations onto its three orthogonal reflections. To perform the hysteresis loop, we started from a relaxed structure at zero electric field that, due to the tensile strain, presented a given buckling of the vortex cores. Afterward, we used the relaxed structure as seed for the next calculation changing the value of the electric field. We used 13 values for the electric field along the [001]*_o_* direction, ranging from −66.82 to +66.82 kV/cm. This procedure was maintained during all the hysteresis loops.

## Supplementary Materials

### This PDF file includes:

Supplementary Text Figs. S1 to S13 Legend for movie S1 References
