# Supplementary material for: Electric field control of chirality
Source: Sci Adv. 2022 Jan 5;8(1):eabj8030. doi: 10.1126/sciadv.abj8030 (PMC8730600; doi:10.1126/sciadv.abj8030)
Supplement: Supplementary file 1 — Supplementary Text Figs. S1 to S13 Legend for movie S1 References [file sciadv.abj8030_sm.pdf]

Science Advances
AAAS

Supplementary Materials for

Electric field control of chirality

Piush Behera, Molly A. May, Fernando Gómez-Ortiz, Sandhya Susarla,
Sujit Das, Christopher T. Nelson, Lucas Caretta, Shang-Lin Hsu, Margaret R. McCarter,
Benjamin H. Savitzky, Edward S. Barnard, Archana Raja, Zijian Hong, Pablo García-Fernandez,
Stephen W. Lovesey, Gerrit van der Laan, Peter Ercius, Colin Ophus, Lane W. Martin,
Javier Junquera*, Markus B. Raschke, Ramamoorthy Ramesh*

*Corresponding author. Email: rramesh@berkeley.edu (R.R.); javier.junquera@unican.es (J.J.)

Published 5 January 2022, *Sci. Adv.* **8**, eabj8030 (2022)
DOI: 10.1126/sciadv.abj8030

The PDF file includes:

Supplementary Text
Figs. S1 to S13
Legend for movie S1
References

Other Supplementary Material for this manuscript includes the following:

Movie S1

Supplementary Text

S1. Different Models of Chirality

Chiral behavior in three dimensions can arise in vortex structures as a consequence of a few symmetry-breaking pathways, which are summarized in Fig. 1 (main text). The first one is the coexistence of vortices with a polarization aligned along the normal of the plane containing the vortex. In $PbTiO_3$/$SrTiO_3$ superlattices, the driving force for the appearance of this axial component can be traced back to the tendency found in common 180° domain walls (DWs) of bulk $PbTiO_3$ to have a Bloch-like character at low temperatures, with a spontaneous electric polarization confined within the DW plane. The DW-DW interaction is so small than the energy split between the parallel and antiparallel configurations of the axial component at neighboring domain walls is negligible. A chiral configuration naturally occurs if an antiparallel axial polarization at the cores of neighboring clockwise/counterclockwise polar tubes condenses, as shown in Fig. 1c.

Even if the axial components align parallel, a second source of chirality can be found if the vortices have non-equal fraction of dipoles pointing along the $[110]_o$-direction (i.e., if the up and down domains are not exactly equal in size) superimposed on an offset of the vortex cores (Fig. 1d). These misalignments immediately yield a mismatch in the polarization pointing along the $[001]_o$-direction (left/right), which generates an excess of the clockwise/counterclockwise rotations. Therefore, for a given size of the domains, the structures adopt a chirality that is dependent on the direction of the mismatch.

The first mechanism described above gives very large values of helicity, since it combines a large value of the rotational component with the coupling with the axial component. Moreover, the contribution of each vortex tube sums in the same direction. However, the switching of chirality would be extremely costly: the two enantiomers would belong to the same topological class and can be transformed into each other in a continuous way. But during this process, some phases with head-to-head and tail-to-tail configurations will inevitably appear, making the path very unlikely, Fig. S3.

The second mechanism produces helicities that are typically two orders of magnitude smaller arising from an imbalance in the rotational component of the two vortices (Figure S2). But it has the advantage that it can be deterministically reversed in a fully controlled way by an external electric field that keeps track of the offset, and therefore the predominant left/right polarization direction, Fig. S1b. Due to the energetic degeneracy in the direction of the axial component, it is likely that left-handed and right-handed domains can coexist in the same sample, Fig. 1e (main text). In such a case, a chiral structure is expected assuming that there exists a mismatch between the axial components combined with the presence of an offset, where the high axial component of the polarization changes from the clockwise to the counterclockwise vortices in adjacent domains as sketched in Fig. 1e.

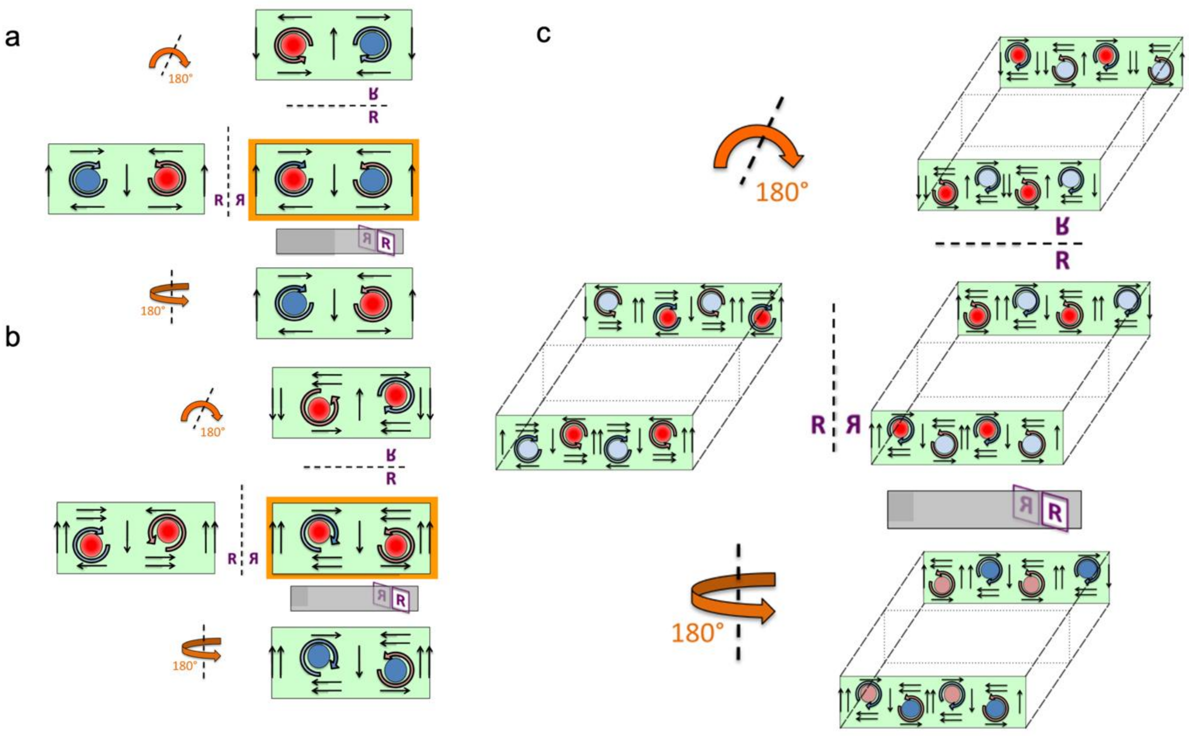

Fig. S1: Key features of the chiral character for the different models of Fig. 1 of the main text. Straight arrows point along the direction of the local polarization.
Clockwise (counterclockwise) vortices are represented by blue (orange) curled arrows. Filled circles represent the direction of the axial component of the polarization, with light colors representing lower values in magnitude. Three orthogonal reflections are examined for each of the models shown in Fig. 1, which are perpendicular to $[001]_o$ (left), $[110]_o$ (upper), and $[1\bar{1}0]_o$ (bottom). Reflections can be mapped onto each other but not to the original one by means of rotations and translations, showing that they are chiral enantiomers.

S2. <u>Origin of chirality in the case of parallel axial components</u>

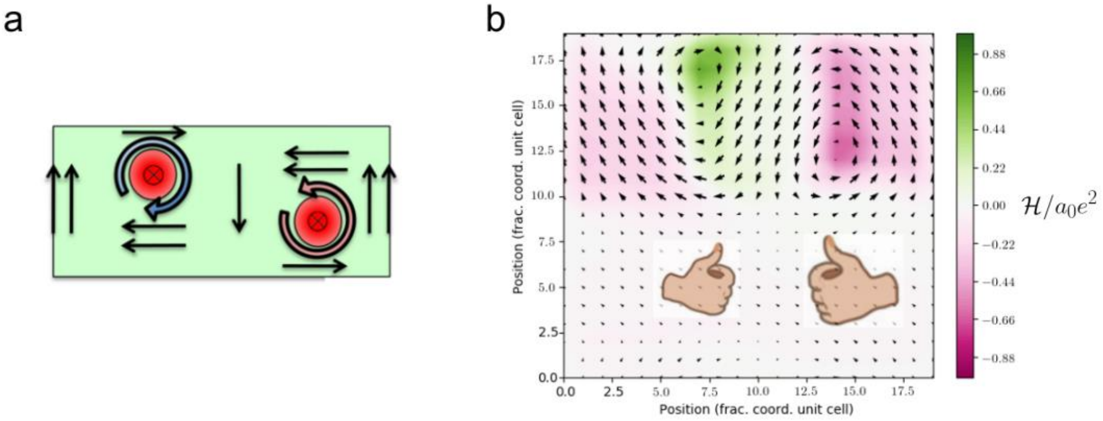

Fig. S2: Origin of the chirality for the vortices with a parallel axial component of the polarization. (A) Sketch of the non-trivial topological texture shown in Fig. 1d. An offset between the center of the vortices is observed along the $[110]_o$-direction, together with a

different size of the up/down domains. The offset leads to a mismatch between the polarization pointing along the $[001]_o$ direction (in the cartoon, the polarization pointing to the left is predominant). The axial component of the polarization is parallel at the center of the two vortices (filled red circles). **(B)** Second-principles results for the same configuration. The arrows represent the in-plane component of the polarization. The colors represent the helicity density (integrand of equation 1), with a scale indicated by the color of the side bar. The handedness of the two vortices is opposite, sketched by the hands in the cartoon. Nevertheless, the combination of a majority of the left-pointing polarization (produced by the offset) with the majority of up polarization yields to the fact that the regions with a negative sign of the helicity (magenta regions) are dominant, making the whole system chiral, as highlighted with the different size of the two hands. In the absence of an offset between the center of the two vortices, there would be no net polarization along $[001]_o$ direction and the previous effect vanishes. If the offset changes the sign, keeping constant the sizes of the up and down domains, then the whole effect reverts its sign, making the right-hand (green regions) predominant.

S3. Pathways to Switch Chirality with Antiparallel Bloch Components in the Domain Wall

When the axial component of the polarization points in opposite directions at the center of the clockwise and counterclockwise vortices, a chiral structure with a large value of the helicity is formed. Both the right- and left-hand enantiomers belong to the same topological class. Therefore, a continuous transition to transform one into the other might be envisaged. Here we propose three different paths, sketched in Fig. S3. The first one (top row in the central panel of Fig. S3) consists of a continuous 180° rotation of all the dipoles that form the vortices. However, there is one midpoint along the path where energetically cost head-to-head and tail-to-tail domains appear. Thus, although topologically allowed, such a transition implies a huge energy barrier, that makes it unlikely. The second mechanism consists in a continuous approximation of the vortex cores, as sketched in the central panel of Fig. S3. But again, the energy barrier to overcome is large: one of the domains along the *z*-direction increases its volume at the expense of the other, and this would translate into large depolarizing fields. Moreover, the Néel-components of the polarization would form head-to-head and tail-to-tail domains. The third path (bottom row in the central panel of Fig. S3) can be described as a homogenous reduction of the axial, Bloch component within the two vortex cores. At some point along the path, the axial component of the polarization would vanish, giving rise to an achiral structure. Beyond this point, a polarization at the center of the vortices opposite to the original one can be developed, changing the sign of the helicity. However, this procedure seems to be impractical, due to the difficulty of applying a spatially dependent external field that change its value in the length-scale of the separation of the vortex cores (around 8 nm).

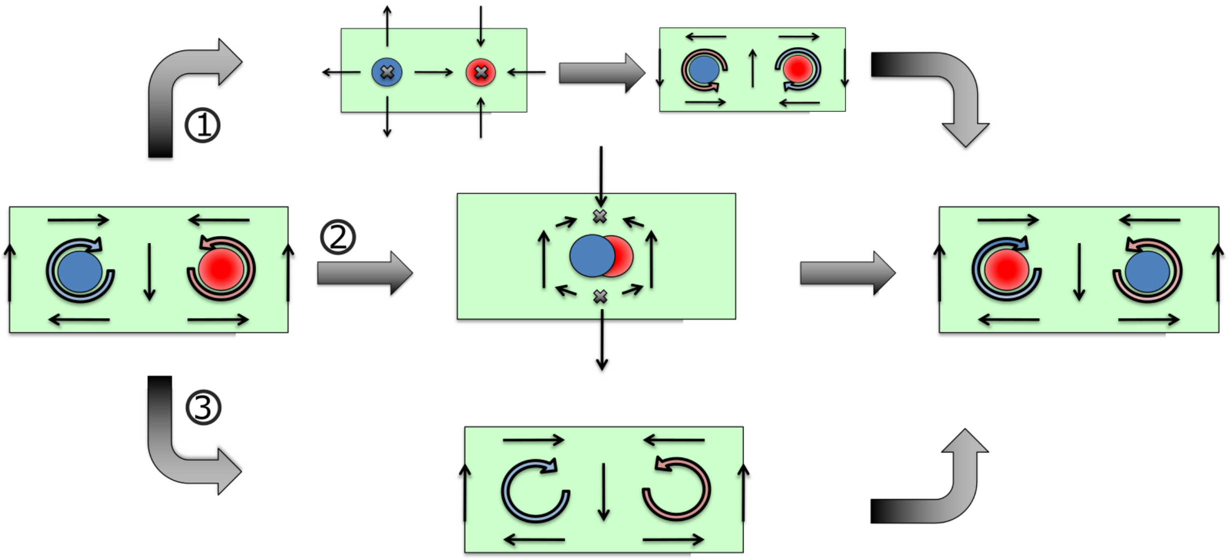

Fig. S3: Switching Pathways. Different paths to switch the helicity of the vortices when the chirality is due to the opposite direction of the axial polarization at the cores of neighbor vortices. Straight arrows represent the direction of the local polarization in the *xz*-plane, while the curled arrows indicate the sense of rotation of the vortices. Red and Blue circles represent different directions of the axial component of the polarization. The crosses are the points where head-to-head and tail-to-tail domains are formed.

S4. Helicity Computation of Chiral Model

Revisiting equation 1, we can observe that two ingredients are necessary to develop helicity. First, we need the curl of the polarization pattern to be non-zero. Second, this curl has to couple with the axial component of the polarization. As discussed in the main body of the manuscript, in the model of Fig. 1e we observe: first an offset between the center of the vortices along the $[110]_o$ direction (responsible for an asymmetry between the polarization components along the $[001]_o$ direction); second, a difference in the sizes of the up and down domains; and third, antiparallel axial components of the polarization at the center of the cores, being one of them slightly larger than the other. In such a model, there are two different sources of chirality. On the one hand, the existence of asymmetries between the UP/DOWN and the LEFT/RIGHT components of the polarization generates an imbalance between the curl of the clockwise and counterclockwise vortices, as shown in the Fig. S2. This is the driving force for the chiral behavior of the structure sketched in Fig. 1d. On the other hand, the anti-parallel axial components of the polarization contributes to the coupling between the curl and the final value of the helicity, responsible for the chirality of the structure discussed in Fig. 1c.(*20*)

Numerically we can assign relative values to both the curl and the axial component. Let us normalize the bare curl contribution to a value of 1 (positive if it is clockwise, and negative it is counter-clockwise). The offset of the center of the cores and the imbalance between the up/down domains induce an increase/decrease of this value by a given amount $\pm\delta$. For instance, in the leftmost tube of the front domain of the sketch of Fig. 1e, a configuration whose curl and domain structure are the same as the one in Fig. S3, the upper clockwise vortex has a curl of $(1-\delta)$, while the down counterclockwise vortex has a curl of $(-1-\delta)$. The fact that the curl is decreased in this case can be deduced from the fact that the negative magenta regions of the curl

are more extended than the positive green region in the Fig. S2. The curls point in the axial direction.

Now, let us also normalize the maximum axial components of the polarization to a value of 1, represented by the dark red circle in the leftmost tube of Fig. 1e. Assuming that this axial component is slightly different in both directions (as schematized in Fig. 1e by the different intensity of the red/blue colors), the axial component in the neighboring vortex takes a value of $\left(-\frac{1}{a}\right)$, were a is a parameter that accounts for the mismatch between components (if $a = 1$, then the two axial components are the same; if $a \to \infty$, one of the axial components vanishes).

Therefore, according to equation 1, the value of the helicity considering the two domains in Fig. 1e is as follows

$$\mathcal{H} \;=\; +1 \cdot (+1 - \delta) - \frac{1}{a}(-1 - \delta) - \frac{1}{a} \cdot (+1 - \delta) + 1(-1 - \delta) = -2\delta + \frac{2\delta}{a}$$

For any value of $a > 1$, i.e. assuming a slight difference in the axial component of the polarization, the helicity of the configuration in Fig. 1e will be negative.

Taking the mirror symmetry of the domain structure of Fig. 1e, as shown in Fig. 1f, the sense of the offset would be reverted, resulting on a change of the curl values that would amount to $(1 + \delta)$ for the clockwise vortices and $(-1 + \delta)$ for the counterclockwise. Computing again the helicity, this would result in a value of $+2\delta - \frac{2\delta}{a}$, the opposite as before, yielding to a change in the handedness.

S5. SHG CD Experimental Details

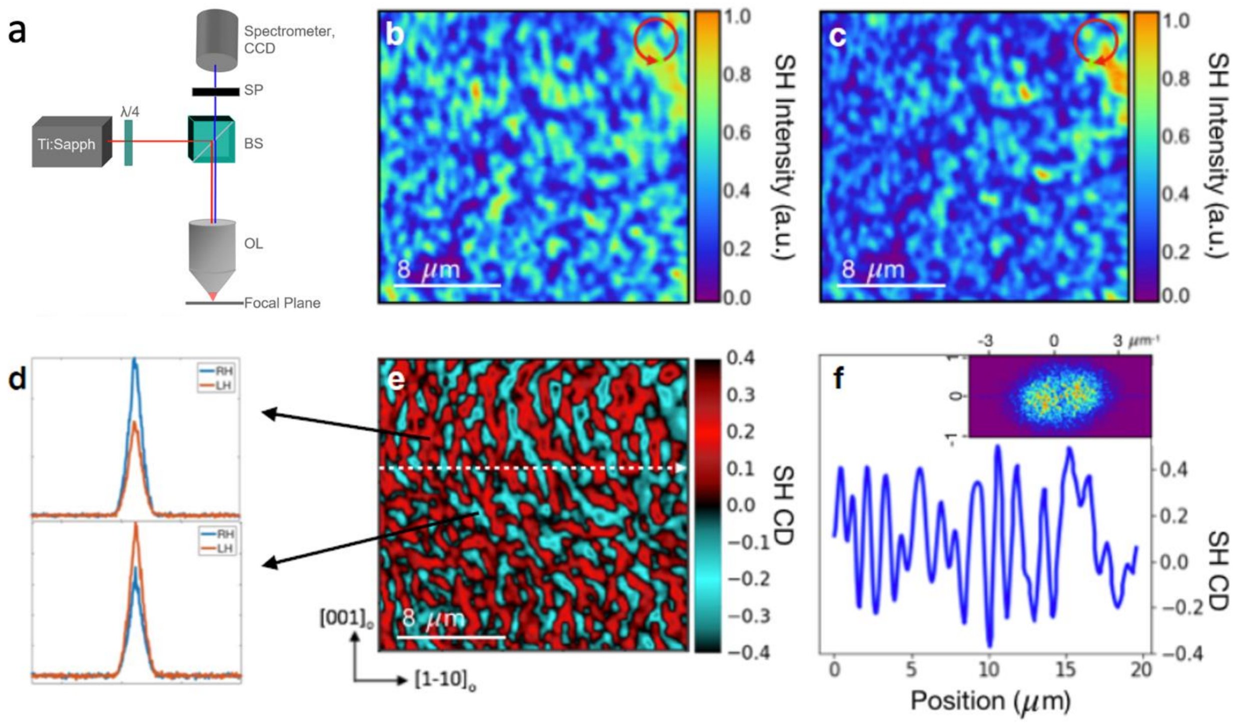

Fig. S4: Experimental Details of SHG-CD. (A) Experimental layout for the SHG-CD measurements. **(B,C)** Examples of SHG images acquired with RC/LC excitation. **(D)** Examples of SHG spectra acquired at a single point in a RC dominant domain (top) and a LC dominant domain (bottom). **(E)** SHG CD calculated from the images shown in (B) and (C). **(F)** Line profile along the dotted line shown in (E) and FFT of the SHG CD showing domain elongation along the $[\mathbf{001}]_{\boldsymbol{o}}$ axis (inset).

SHG-CD measurements were made as described in the methods section and depicted in Fig. S4a. To perform an SHG-CD measurement, the integrated intensity of the SHG signal at a single location on the sample was measured using RC polarized excitation light, producing a response with intensity I_{RH}.Next, the intensity was measured at the same location using LC polarized excitation to find I_{LH}. and the CD signal was calculated. Fig. S4b,c shows examples of SHG imaging with RC and LC excitation and Fig. S4d shows example spectra acquired in a RC domain (top) and an LC domain (bottom). Fig. S4e shows a 2D map of the SHG CD corresponding to the many integrated spectral pairs in parts **e** and **c** calculated using equation 2 in the main text. The characteristic length scale is shown by the line profile in Fig. S5f with a FFT of the image shown in the inset revealing domain elongation along the $[001]_o$ axis.

To further validate the SHG-CD measurements we performed a series of control experiments outlined in Fig. S5. First, we show an SHG-CD image (5c) along with the LC (5a) and RC (5b) images for comparison. Next, we measured SHG maps with 0° (5d) and 90°(5e) linearly polarized excitation, corresponding to polarizations along the $[001]_o$ and $[1\bar{1}0]_o$ sample axes, respectively. From these, we calculated the SHG linear dichroism (LD) shown in 6f using the

equation $LD = \frac{I_{90^\circ} - I_{0^\circ}}{I_{90^\circ} + I_{0^\circ}}$. This reveals LD that is much weaker than the observed CD, confirming that the CD signal cannot simply be an artifact related to large LD anisotropy. In contrast to the chiral domains composed of polar vortices, we have measured SHG CD for the traditional ferroelectric *a*-domains which form in a $(PTO)_n/(STO)_n$ trilayer heterostructure with $n = 8$ as an achiral control structure. We observe negligible CD signal for this sample, on the order of 10% of that observed in the $n = 20$ sample, as shown in (g)-(j), indicating that the CD signal is indeed related to polar domain formation. Finally, we confirmed that we are creating pure LC and RC light by comparing the normalized sum of the LC and RC excitation images (5j) to an image taken with linear excitation (5k). These images should look the same because linear polarized light is a superposition of LC and RC polarized light and thus excites both domain types equally. While some variation exists, these images agree to within the expectation. It is worth noting that the color maps for the calculated images shown in 6c, 5f, and 5j have been normalized to the same values so that their intensities can be directly compared.

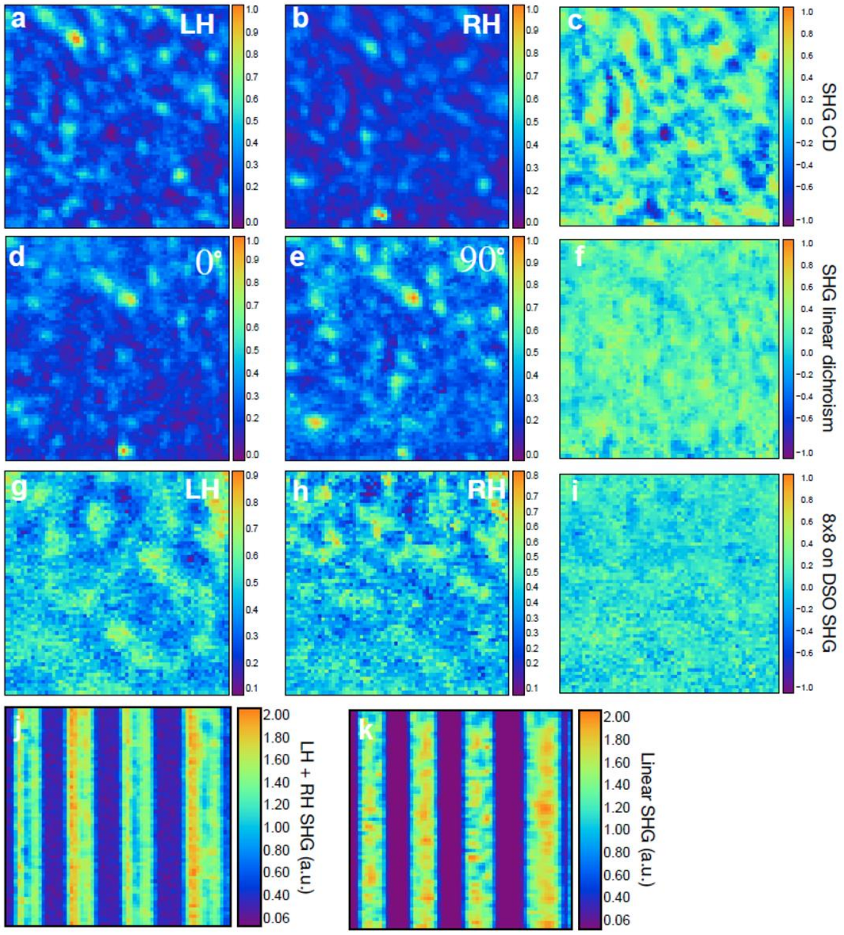

Fig. S5: Validation of SHG-CD Results. (A-C) LC and RC SHG images and calculated CD for a normal sample region. d-f, Linear dichroism for the same region. **(G-I)** SHG CD for a ferroelectric trilayer structure with no vortex formation. **(J-K)** Sum of LC and RC images, and linear SHG showing that imaging with linear excitation excites both LC and RC modes.

S6. Origins of Second Harmonic Generation

Second harmonic generation is a photonic exchange between the frequency components of the electromagnetic field. During the interaction with matter, two photons of lower frequency, ω ,are absorbed and one photon of 2ω is created in a single quantum-mechanical process. Mathematically, an incident light source will interact with a medium's electronic structure, displacing the charged particles in the material during a process called polarization. Linear materials become polarized to a degree proportional to the intensity of the incident electric field

$$\mathbf{P} = \varepsilon_0 \chi^{(1)} \mathbf{E} \qquad \text{(S1)}$$

where $\chi^{(\mathbf{1})} = n^{\mathbf{2}} - 1$ is the linear susceptibility tensor for refractive index n which reflects the polarizability along each axis of the material. For very intense driving fields, however, nonlinearities in the material can be probed. The nonlinear polarization can be expressed as the Taylor expansion of the material's polarization in powers of the electric field with each component of **P** expressed as P_k where $k = x, y, z$:

$$\mathbf{P} = \varepsilon_0 (\chi_{ik}^{(1)} \mathbf{E}_{\boldsymbol{i}} + \chi_{ijk}^{(2)} \mathbf{E}_{\boldsymbol{i}} \mathbf{E}_{\boldsymbol{j}} + \chi_{ijlk}^{(3)} \mathbf{E}_{\boldsymbol{i}} \mathbf{E}_{\boldsymbol{j}} \mathbf{E}_{\boldsymbol{l}} + \ldots) \qquad \text{(S2)}$$

where the coefficients of $\chi^{(n)}$are tensors of increasing rank which correspond to the nth order process. These tensors describe the susceptibilities of the material along each axis and also coupling between material axes. The higher order processes are so weak that they were predicted but never observed until the advent the lasers in the 1960s(*49*).

Second harmonic generation arises from symmetry breaking in the second order susceptibility, which corresponds to a rank 3 tensor that describes a material's susceptibility along each axis. The second order polarizability goes as the electric field squared and for an incident electric field $E_i = \varepsilon_i e^{-i\omega t} + c.c$, it can be represented as

$$\mathbf{P}_{\boldsymbol{k}}(\boldsymbol{NL}) = \chi_{ijk}^{(2)} (\varepsilon_i \varepsilon_j e^{-i2\omega t} + \varepsilon_i^* \varepsilon_j^* e^{i2\omega t} + \varepsilon_i \varepsilon_j^* + \varepsilon_i^* \varepsilon_j) \qquad \text{(S3)}$$

From this equation, it can easily be shown that frequency doubling, or SHG, of the incident light is allowed.

Second-harmonic generation is sensitive to symmetry breaking, which can be seen by applying a spatial inversion operation to equation S3. The parity operation which takes $r \to -r$ changes the sign of both the polarization and electric fields. However, since the second order polarization goes as the square of the electric field, this term remains positive unless $\chi_{ijk}^{(2)}$, the susceptibility tensor, changes sign with the inversion. Only materials with susceptibility tensors that are sensitive to spatial inversion have a nonzero second order response. Physically this means that the material polarizes differently along different axes, and materials with this trait are commonly

called noncentrosymmetric. This sensitivity to inversion symmetry makes second-harmonic generation a powerful tool for imaging crystal lattices with symmetry breaking, which often occurs at domain boundaries(*30*).

S7. Circular Dichroism in Second Harmonic Generation

A SHG microscope can selectively observe the region in a sample where spatial inversion symmetry is broken. The attribute′s puissance has fueled a surge of applications of the technique to advance knowledge about nanostructures, molecular ordering, and structural organization in biological samples. Furthermore, the advent of free-electron lasers in the energy ranges from extreme ultraviolet to x-rays now allows to explore SHG effects involving core-level resonances.

A sophisticated set of theoretical tools at the disposal of exponents of ordinary NCD has been granted to users of NCD in the SHG response(*35*, *50*). To begin with, ordinary dichroic signals use dyadic matrix-elements of the light-matter interaction found in the familiar Kramers-Heisenberg amplitude that is trivially extended to parity-odd E1-E2 or E1-M1 scattering events to cope with NCD(*51*). Insight into the matrix elements is achieved by integrating out intermediate degrees of freedom, in the footsteps of Judd and Ofelt in their celebrated work on optical transition probabilities(*52*, *53*). Specifically, a dyadic matrix element in the interaction is reduced to a spectrum of electronic multipoles routinely exploited in analyses and simulations of data gathered by other experimental techniques, including, NMR, EPR, Mössbauer effect, and resonant x-ray Bragg diffraction. Equivalent benefits accrue in an application of similar mathematical treatment to the NCD signal in the SHG response. In this case, a parity-odd E1′-E1-E1 process contains an NCD signal when angular momentum (**L**) and space (**R**) know inextricable knots which bind each to the other in the illuminated sample (E1 primary and E1′ secondary photon events)(*35*). On the other hand, no NCD is allowed for the nominally weaker E1′-M1-M1 process.

A third-order perturbation theory accounts for the SHG response. The generic form of the NCD signal from the E1′-E1-E1 response is,

$$F(NCD)\ P_2 \sum_{\lambda,\lambda'} \{\langle g|x|\lambda\rangle\langle\lambda|y|\lambda'\rangle\langle\lambda'|z|g\rangle - \langle g|y|\lambda\rangle\langle\lambda|x|\lambda'\rangle\langle\lambda'|z|g\rangle\} \qquad \text{(S4)}$$

Here, coordinates are defined by a primary beam parallel to the z-axis and σ-polarization parallel to x-axis, and the electric dipole $\mathbf{R} = (x, y, z)$. The secondary wavevector, for E1′, is inclined to the z-axis and its polarization vector casts a shadow on the axis. P_2 is the pseudo-scalar Stokes parameter for circular polarization. Labels λ, λ' delineate intermediate degrees of electronic freedom, while g denotes the ground-state.

The act in equation S4 of integrating out λ, λ' creates atomic multipoles $\mathbf{U^K}$, where K is the rank. The expectation value of the dipole $\langle\mathbf{U^1}\rangle$ is the electric polarization in the electronic ground-state, for example. More generally, multipoles form observable signals that must be purely real, and the requirement restricts the composition of $\mathbf{U^K}$. For any process, such as E1′-E1-E1, there is a conjugate process that assures the response is purely real. Additional restrictions, or selection rules, flow from integrating out λ, λ'. Where upon, E1′-E1-E1 is no longer a simple product of

three matrix elements of **R**. Rather, dipole matrix elements interlock in a very specific manner that reveals the nature of the SHG response, notably, selective replacement of **R** by **L**. One component of it remains after the fine sieve of photon polarization and the component in NCD is the quadrupole $\langle \mathbf{U}^2 \rangle$. Results for the SHG response are energy integrated signals. That is to say, from Eq. (8), $\langle U^2 \rangle \propto \{\mathbf{R} \otimes \mathbf{C}^2(\mathbf{R})\}^2$ is the total NCD signal available from a substance in a suitably designed measurement. Here, $\{.\otimes .\}^K$ denotes a standard tensor product of rank K, $\mathbf{C}^2(\mathbf{R})$ is a spatial spherical harmonic of rank 2 normalized such that $\mathbf{C}^1(\mathbf{R}) = \mathbf{R}$. The corresponding energy-integrated MCD signal is a correlation of orbital angular momentum and space, specifically, a sum of multipoles $\{\mathbf{C}^2(\mathbf{R}) \otimes \mathbf{L}\}^K$ with $K = 1$ and 3 (*36*).

S8. 4D-STEM

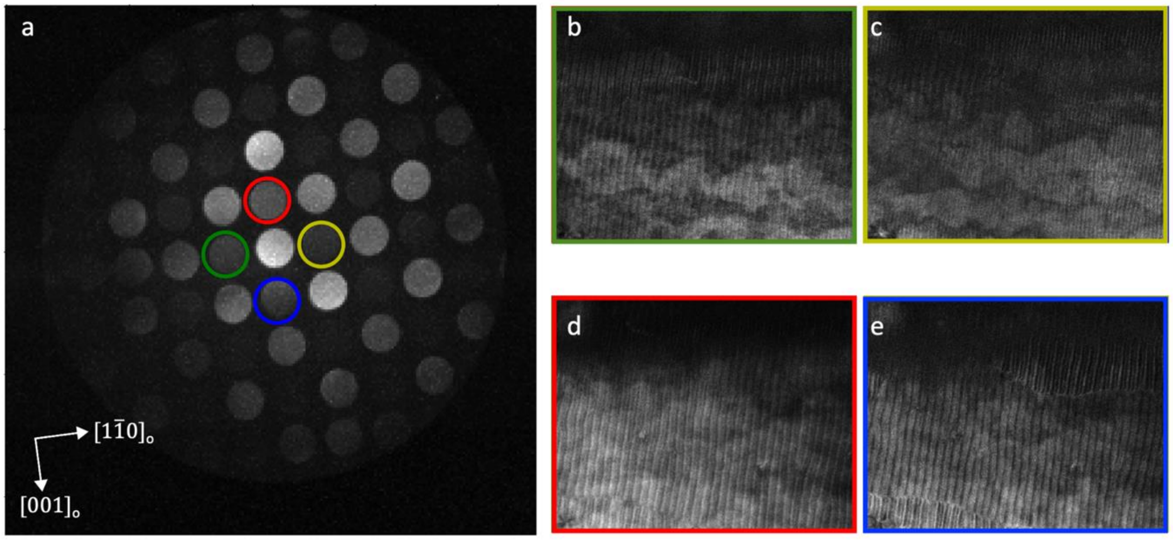

Fig. S6: Example of 4D-STEM Analysis. (A) Normalized CBED pattern extracted from entire region in Figure 3a. **(B-E)** Virtual images that are formed by selected the different diffraction disks along $[001]_o$ (red/blue) and $[1\bar{1}0]_o$ (green/yellow) directions.

The 4D STEM data was analyzed using py4dstem software package(*54*). Prior to determining the polarization from the diffraction patterns, the diffraction disks were corrected for diffraction shift and scan coil rotation in order to deterministically locate the $[001]_o$and $[1\bar{1}0]_o$directions. After all the calibrations, Bragg disks were detected on diffraction pattern at every scan position. Then two Friedel pairs, Fig. S6a, in the $[1\bar{1}0]_o$ direction (green and yellow circle) and $[001]_o$ direction (red and blue circle) was selected to generate the respective virtual images, Fig. S6d-e. The polarization was determined by subtracting the normalized intensity difference between virtual images formed by the red and blue circles for lateral or $[001]_o$ polarization and green and yellow circles for axial polarization $[1\bar{1}0]_o$.

S9. Electric Field Dependent Measurements

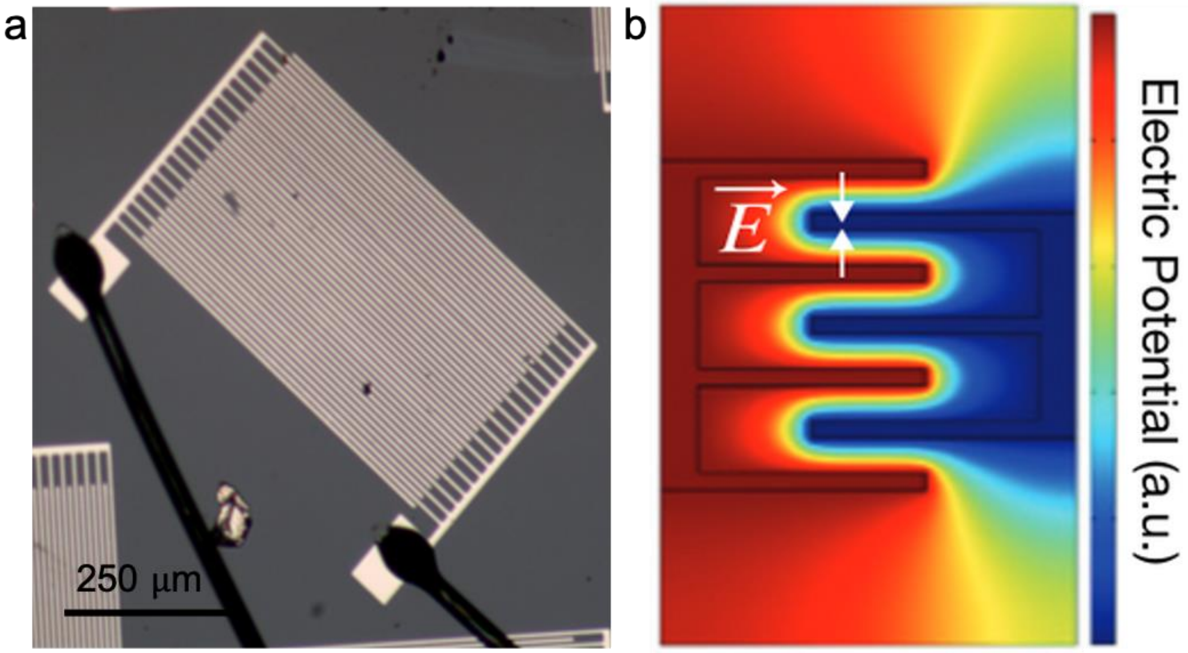

Fig. S7: Structure of Interdigitated Electrodes. (A) Image of representative IDE device used. **(B)** Calculated electric potential for and IDE device, demonstrating opposite electric field across neighboring fingers.

For electric field measurements interdigitated electrodes were deposited on the sample surface (Fig. S7a). These electrodes create constant electric field with alternating sign across neighboring interdigitated regions.

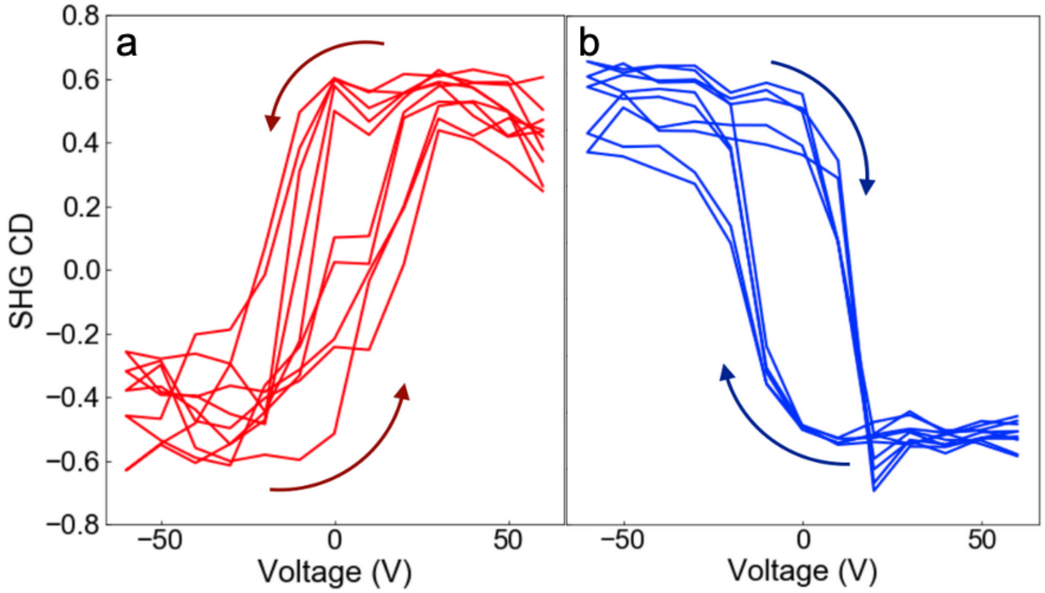

Fig. S8: SHG-CD Hysteresis. (A-B) Hysteresis repeated in five different RC and LC oriented chiral domains, respectively, which show the expected mirrored behavior in the CD signal.

The phase change between LC and RC chirality was characterized by measuring its hysteresis. This was done as described in the main text by recording the SHG CD for applied voltages starting at +60 V, decreasing in increments of 10 V to -60 V, and reversing the process for increasing voltages back to +60 V. This revealed clear hysteresis behavior as shown in Fig. S8a. Furthermore, this behavior was observed to follow the inverted pattern shown in Fig. S8b as expected for regions of opposite applied field. These hysteresis measurements were repeated at several sample locations with good reproducibility as shown by the data in Fig. S8 for regions of both field polarities.

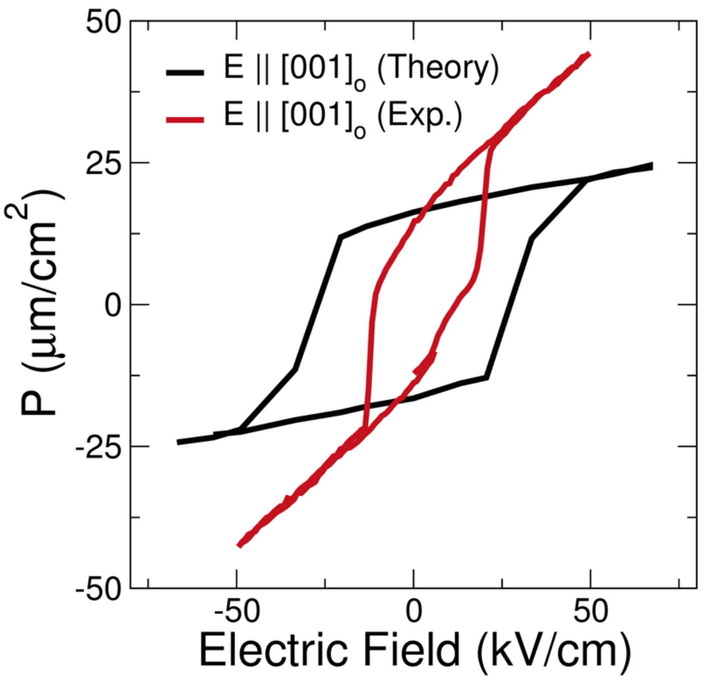

Fig. S9: Hysteresis Loops. Raw data for polarization vs. electrical field measurements performed with the electric field along the $[001]_o$ axis (red). Polarization response from second principles is seen in black.

In-plane capacitance measurements using similar devices to those used for Fig. S7a, yield the polarization vs. electric field data presented in Fig S9, in red. The large dielectric contribution was subtracted when presented in Fig. 4e. A similar procedure was followed for the polarization calculated from second principles (in black), where the lesser dielectric contribution was subtracted out when presented in Fig. 4e.

S10. Second Principles Simulation

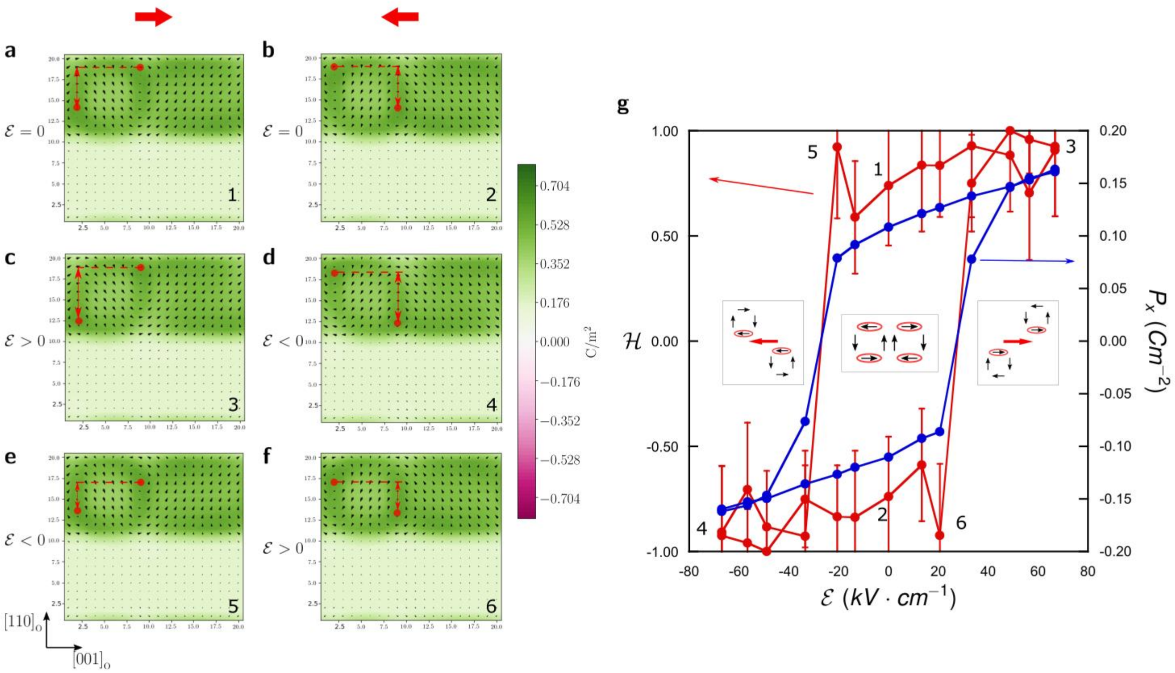

Fig. S10: Switching of the chirality due to buckling. (A-B) Enantiomer configurations of vortices with opposite buckling. Filled red points indicate location of vortex cores. Large red arrows at the top indicate direction of net polarization along $[001]_o$ induced by the offset. **(C-D)** Displacement of the vortices when the field is applied parallel to the net in-plane polarization. The vertical separation between the vortex cores is enhanced. **(E-F)** Displacement of the vortices when the field is applied anti-parallel to net in-plane polarization previous to the coercive field. The vertical distance between the vortex cores is progressively reduced. **(G)** Helicity (red; left axis) and polarization (blue; right axis) for model system as a voltage is applied along $[001]_o$. Numbering of points relates numerical values of the helicity to polarization patterns presented in (A-F). Insets schematize the direct coupling between the sense of the buckling and the $[001]_o$ component of the polarization.

S11. In-Situ DF-TEM Studies

By changing the reflection conditions used in DF-TEM, we are able to probe the orientation of domains within the vortex phase. Focusing on the $[110]_o$ family of reflections, gives rise to contrast along the c-axis. This allows for differentiating between UP/DOWN domains, as seen in Fig. 4a-c and Fig. S11. Using the $[001]_o$, allows for differentiating between domains with opposing $[001]_o$ polarization, Fig. S11b. The combination of the two allows for the assignment of vectoral polarization depending on the intensity seen in DF-TEM.

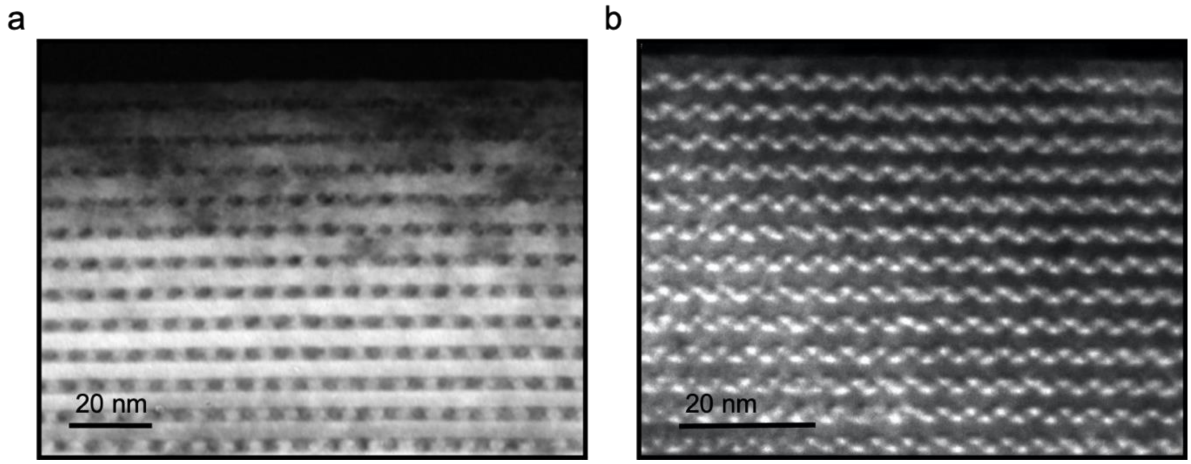

Fig. S11: DF-TEM Analysis. (A) DF-TEM image taken using the $[220]_o$ reflection, giving rise to contrast from domains with opposing displacement along the *c*-axis. **(B)** DF-TEM image taken using $[008]_o$ reflection, giving rise to contrast from domains with opposing displacement along the *a*-axis

Application of a tip on the surface of the cross-sectioned sample allows for probing of both in-plane and out-of-plane polarization. Fig. S12 illustrates that depending on the location of the center of the tip relative to the sample, there will be an increased in-plane field across the sample the further away from the tip center one images. The attached Movie S1 shows the transformation of the vortices from both a majority out-of-plane and majority in-plane polarization.

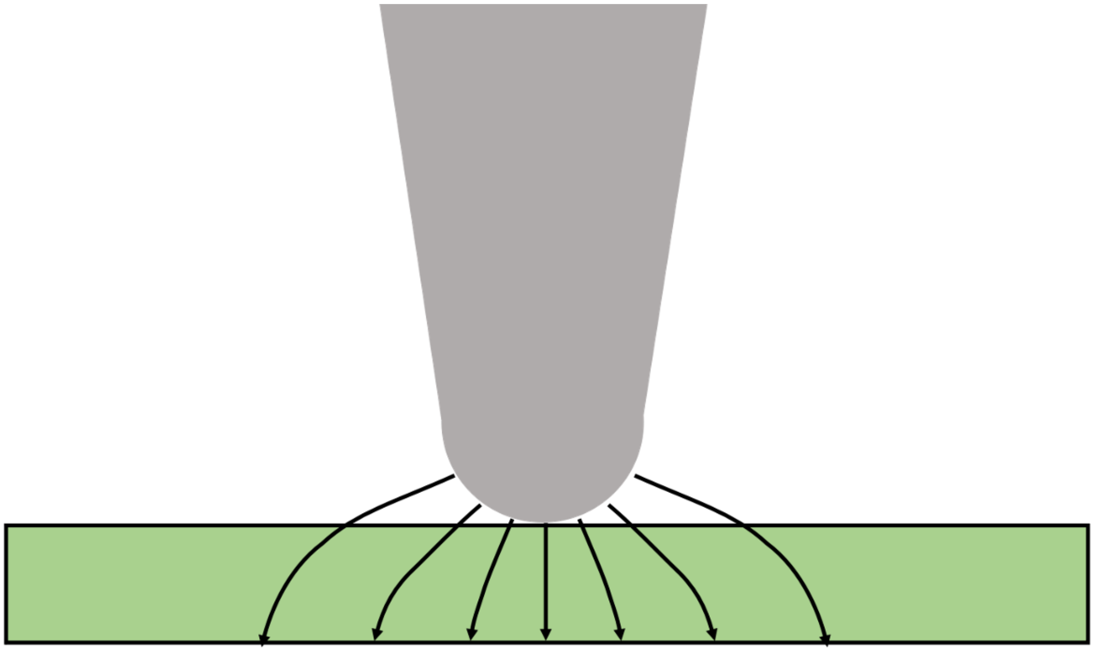

Fig. S12: Schematic of *in* situ application of electric fields in DF-TEM. Schematic of electric field applied through a tip. Demonstrates mixture of in-plane and out-of-plane fields.

DF-TEM data for intermediate applied voltages throughout the chiral phase transition is provided in Fig. S13. Images were taken at increments of ~1.8V, with the majority of the field along the $[001]_o$ in-plane direction. The first transition is seen going from 3.7 to 5.6V, where the net in-plane polarization switching causing a reversal in the vortex buckling. As the voltage increases, the system approached a pure ferroelectric phase seen at 15.0V. This further demonstrates that the majority of the field lies in-plane for this location. A video (Movie S1) of the transition has also been provided, which demonstrates reversibility of the chiral phase transition.

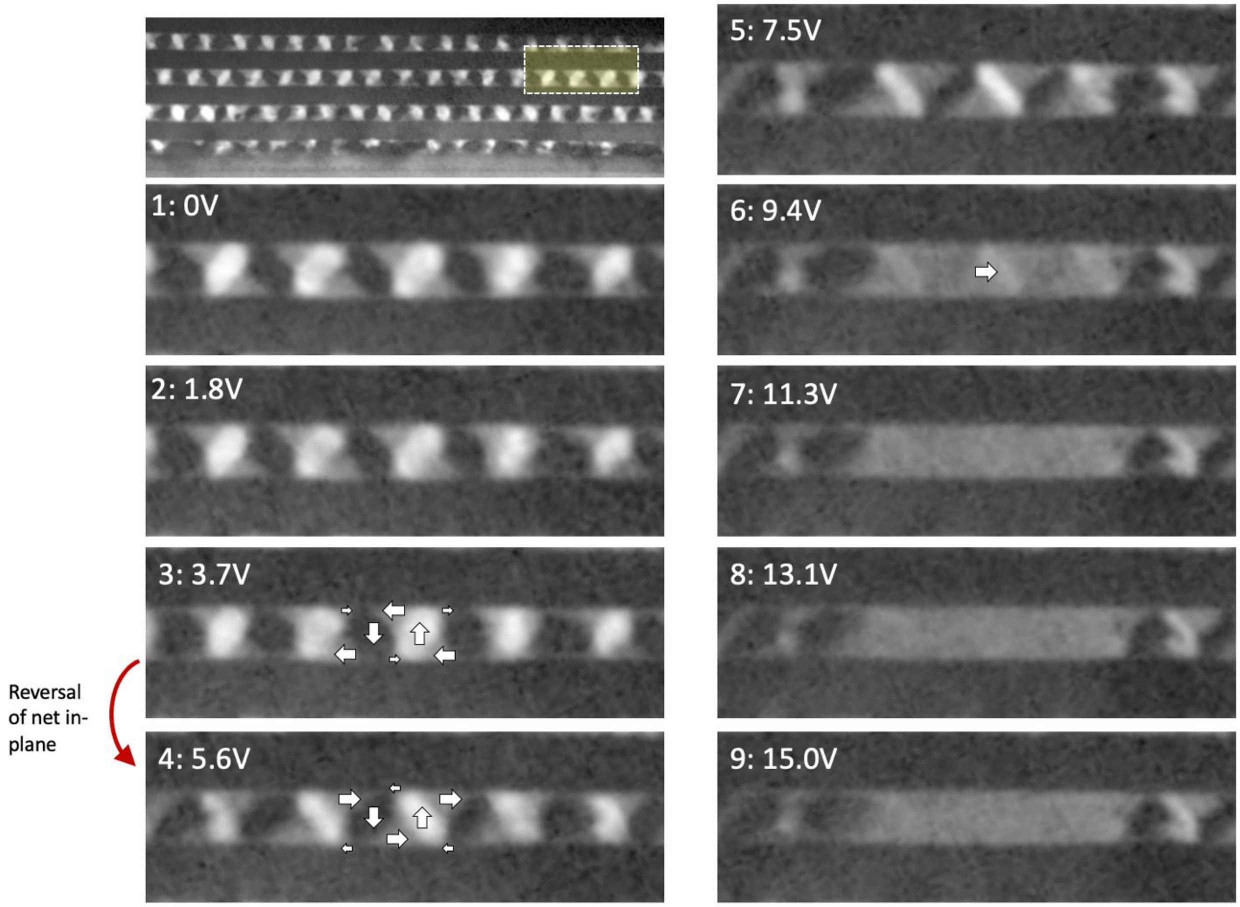

Fig. S13: DF-TEM imaging of chiral phase transition. DF-TEM images for increasing applied voltages showing microscopic restructuring across the in-plane reversal of polarization and transition to pure ferroelectric state at higher voltages

S12. Chirality in Ferroic Materials

Chirality is a widespread phenomenon in nature that indicates symmetry breaking in which an object's mirror image cannot be superimposed onto the original object. The chirality of a particle can have a profound effect on its behavior. For example, while all glucose molecules taste sweet, the human body can only metabolize naturally occurring glucose molecules, which have a right-handed chirality. On the other hand, glucose engineered by humans to have a left-handed chirality cannot be metabolized and is commonly used as a calorie-free sweetener.

Chiral phenomena in ferroic materials such as chiral magnetic domains(*55*) and chirality in nanoscale magnets(*56*) have been recognized as a promising path to extending Moore's Law for decreasing the spatial extent and energy consumption of information storage and processing devices using spintronics. In these materials, the spin-orbital interaction, called the Dzyaloshinskii-Moriya interaction, twists the magnetization to induce topological defects such as chiral magnetic, and topological chiral domain walls(*57*, *58*). Additionally, the ability to tune the chirality of Bloch-type and Néel-type domain walls has been demonstrated(*6*, *59*, *60*) as an important step toward the control necessary to read and write spin-based bits.

However, domains in ferroelectric materials are usually separated by domain walls with uniform polarization and no significant chirality(*61*). Complex ferroelectric domain walls such as Bloch-type and Néel- type domain walls are associated with local polarization rotation and offer a potential path to discovering chirality in ferroelectric materials(*62*, *63*). However, topological defects such as ferroelectric vortex structures are generally considered the most promising candidates for applications in technology due to their ability to support clockwise (CW) and counterclockwise (CCW) rotating polarizations. For ferroelectric materials with CW and CCW polarization rotations in addition to an axial polarization component, it has been shown theoretically that the 3D vortex state can possess chirality with different handedness(*64*).

In recent studies, polar vortices in ferroelectric superlattices were created(*13*, *65*) by tuning the interactions between the electrostatic and elastic boundary conditions through the layer thickness, substrate, and choice of materials. In these materials, polar vortices form in ordered arrays with continuous rotation of the polarization and coexist with ferroelectric a-domains(*22*). A significant discovery recently showed that polar vortices in $PbTiO_3$/$SrTiO_3$ superlattices exhibit strong circular dichroism when circularly polarized light is used in resonant soft X-ray diffraction, indicating that these vortices may have a chiral structure(*20*).

Movie S1: *In Situ* DF-TEM study of vortex response to an applied field.

In-situ DF-TEM movie of vortex response to external out-of-plane **(Top)** and in-plane **(Bottom)** electric field.

REFERENCES AND NOTES

1. B. B. V. Aken, J.-P. Rivera, H. Schmid, M. Fiebig, Observation of ferrotoroidic domains. *Nature* **449**, 702–705 (2007).

2. N. A. Spaldin, M. Fiebig, M. Mostovoy, The toroidal moment in condensed-matter physics and its relation to the magnetoelectric effect. *J. Phys. Condens. Matter* **20**, 434203 (2008).

3. Y. Nambu, Nobel Lecture: Spontaneous symmetry breaking in particle physics: A case of cross fertilization. *Rev. Mod. Phys.* **81**, 1015–1018 (2009).

4. N. K. Richtmyer, C. S. Hudson, The oxidative degradation of L-glucoheptulose. *J. Am. Chem. Soc.* **64**, 1609–1611 (1942).

5. W. Sowa, Synthesis of L-glucurone. Conversion of D-glucose into L-glucose. *Can. J. Chem.* **47**, 3931–3934 (1969).

6. G. Chen, A. T. N'Diaye, S. P. Kang, H. Y. Kwon, C. Won, Y. Wu, Z. Q. Qiu, A. K. Schmid, Unlocking Bloch-type chirality in ultrathin magnets through uniaxial strain. *Nat. Commun.* **6**, 6598 (2015).

7. M. Nakata, R.-F. Shao, J. E. Maclennan, W. Weissflog, N. A. Clark, Electric-field-induced chirality flipping in smectic liquid crystals: The role of anisotropic viscosity. *Phys. Rev. Lett.* **96**, 067802 (2006).

8. X. Yin, Z. Ye, J. Rho, Y. Wang, X. Zhang, Photonic spin Hall effect at metasurfaces. *Science* **339**, 1405–1407 (2013).

9. A. O. Govorov, Y. K. Gun'ko, J. M. Slocik, V. A. Gérard, Z. Fan, R. R. Naik, Chiral nanoparticle assemblies: Circular dichroism, plasmonic interactions, and exciton effects. *J. Mater. Chem.* **21**, 16806–16818 (2011).

10. M. Li, L.-J. Chen, Y. Cai, Q. Luo, W. Li, H.-B. Yang, H. Tian, W.-H. Zhu, Light-driven chiral switching of supramolecular metallacycles with photoreversibility. *Chem* **5**, 634–648 (2019).

11. I. I. Naumov, L. Bellaiche, H. Fu, Unusual phase transitions in ferroelectric nanodisks and nanorods. *Nature* **432**, 737–740 (2004).

12. A. Gruverman, D. Wu, H.-J. Fan, I. Vrejoiu, M. Alexe, R. J. Harrison, J. F. Scott, Vortex ferroelectric domains. *J. Phys. Condens. Matter* **20**, 342201 (2008).

13. A. K. Yadav, C. T. Nelson, S. L. Hsu, Z. Hong, J. D. Clarkson, C. M. Schlepütz, A. R. Damodaran, P. Shafer, E. Arenholz, L. R. Dedon, D. Chen, A. Vishwanath, A. M. Minor, L. Q. Chen, J. F. Scott, L. W. Martin, R. Ramesh, Observation of polar vortices in oxide superlattices. *Nature* **530**, 198–201 (2016).

14. X. Z. Yu, N. Kanazawa, Y. Onose, K. Kimoto, W. Z. Zhang, S. Ishiwata, Y. Matsui, Y. Tokura, Near room-temperature formation of a skyrmion crystal in thin-films of the helimagnet FeGe. *Nat. Mater.* **10**, 106–109 (2011).

15. S. Seki, X. Z. Yu, S. Ishiwata, Y. Tokura, Observation of skyrmions in a multiferroic material. *Science* **336**, 198–201 (2012).

16. N. Nagaosa, Y. Tokura, Topological properties and dynamics of magnetic skyrmions. *Nat. Nanotechnol.* **8**, 899–911 (2013).

17. Y. Tokunaga, X. Z. Yu, J. S. White, H. M. Rønnow, D. Morikawa, Y. Taguchi, Y. Tokura, A new class of chiral materials hosting magnetic skyrmions beyond room temperature. *Nat. Commun.* **6**, 7638 (2015).

18. Y. L. Tang, Y. L. Zhu, X. L. Ma, A. Y. Borisevich, A. N. Morozovska, E. A. Eliseev, W. Y. Wang, Y. J. Wang, Y. B. Xu, Z. D. Zhang, S. J. Pennycook, Observation of a periodic array of flux-closure quadrants in strained ferroelectric $PbTiO_3$ films. *Science* **348**, 547–551 (2015).

19. Y. J. Wang, Y. P. Feng, Y. L. Zhu, Y. L. Tang, L. X. Yang, M. J. Zou, W. R. Geng, M. J. Han, X. W. Guo, B. Wu, X. L. Ma, Polar meron lattice in strained oxide ferroelectrics. *Nat. Mater.* **19**, 881–886 (2020).

20. P. Shafer, P. García-Fernández, P. Aguado-Puente, A. R. Damodaran, A. K. Yadav, C. T. Nelson, S.-L. Hsu, J. C. Wojdeł, J. Íñiguez, L. W. Martin, E. Arenholz, J. Junquera, R. Ramesh, Emergent chirality in the electric polarization texture of titanate superlattices. *Proc. Natl. Acad. Sci. U.S.A.* **115**, 915–920 (2018).

21. A. K. Yadav, K. X. Nguyen, Z. Hong, P. García-Fernández, P. Aguado-Puente, C. T. Nelson, S. Das, B. Prasad, D. Kwon, S. Cheema, A. I. Khan, C. Hu, J. Íñiguez, J. Junquera, L.-Q. Chen, D. A. Muller, R. Ramesh, S. Salahuddin, Spatially resolved steady-state negative capacitance. *Nature* **565**, 468–471 (2019).

22. A. R. Damodaran, J. D. Clarkson, Z. Hong, H. Liu, A. K. Yadav, C. T. Nelson, S.-L. Hsu, M. R. McCarter, K.-D. Park, V. Kravtsov, A. Farhan, Y. Dong, Z. Cai, H. Zhou, P. Aguado-Puente, P. García-Fernández, J. Íñiguez, J. Junquera, A. Scholl, M. B. Raschke, L.-Q. Chen, D. D. Fong, R. Ramesh, L. W. Martin, Phase coexistence and electric-field control of toroidal order in oxide superlattices. *Nat. Mater.* **16**, 1003–1009 (2017).

23. L. Louis, I. Kornev, G. Geneste, B. Dkhil, L. Bellaiche, Novel complex phenomena in ferroelectric nanocomposites. *J. Phys. Condens. Matter* **24**, 402201 (2012).

24. J.-Y. Chauleau, T. Chirac, S. Fusil, V. Garcia, W. Akhtar, J. Tranchida, P. Thibaudeau, I. Gross, C. Blouzon, A. Finco, M. Bibes, B. Dkhil, D. D. Khalyavin, P. Manuel, V. Jacques, N. Jaouen, M. Viret, Electric and antiferromagnetic chiral textures at multiferroic domain walls. *Nat. Mater.* **19**, 386–390 (2019).

25. S. W. Lovesey, G. van der Laan, Resonant x-ray diffraction from chiral electric-polarization structures. *Phys. Rev. B* **98**, 155410 (2018).

26. W. J. Chen, S. Yuan, L. L. Ma, Y. Ji, B. Wang, Y. Zheng, Mechanical switching in ferroelectrics by shear stress and its implications on charged domain wall generation and vortex memory devices. *RSC Adv.* **8**, 4434–4444 (2018).

27. L. L. Ma, Y. Ji, W. J. Chen, J. Y. Liu, Y. L. Liu, B. Wang, Y. Zheng, Direct electrical switching of ferroelectric vortices by a sweeping biased tip. *Acta Mater.* **158**, 23–37 (2018).

28. Y. Tikhonov, S. Kondovych, J. Mangeri, M. Pavlenko, L. Baudry, A. Sené, A. Galda, S. Nakhmanson, O. Heinonen, A. Razumnaya, I. Luk'yanchuk, V. M. Vinokur, Controllable skyrmion chirality in ferroelectrics. *Sci. Rep.* **10**, 8657 (2020).

29. P. Chen, C. Tan, Z. Jiang, P. Gao, Y. Sun, X. Li, R. Zhu, L. Liao, X. Hou, L. Wang, K. Qu, N. Li, X. Li, Z. Xu, K. Liu, W. Wang, J. Wang, X. Ouyang, X. Zhong, J. Wang, X. Bai, Manipulation of polar vortex chirality in oxide superlattices. arXiv:2104.12310 [cond-mat.mtrl-sci] (26 April 2021).

30. R. W. Boyd, *Nonlinear Optics* (Academic Press, 2020).

31. P. Fischer, F. Hache, Nonlinear optical spectroscopy of chiral molecules. *Chirality* **17**, 421–437 (2005).

32. T. Petralli-Mallow, T. M. Wong, J. D. Byers, H. I. Yee, J. M. Hicks, Circular dichroism spectroscopy at interfaces: A surface second harmonic generation study. *J. Phys. Chem.* **97**, 1383–1388 (1993).

33. X. Yin, M. Schäferling, B. Metzger, H. Giessen, Interpreting chiral nanophotonic spectra: The plasmonic Born–Kuhn model. *Nano Lett.* **13**, 6238–6243 (2013).

34. A. Belardini, A. Benedetti, M. Centini, G. Leahu, F. Mura, S. Sennato, C. Sibilia, V. Robbiano, M. C. Giordano, C. Martella, D. Comoretto, F. B. de Mongeot, Second harmonic generation circular dichroism from self-ordered hybrid plasmonic–photonic nanosurfaces. *Adv. Opt. Mater.* **2**, 208–213 (2014).

35. S. W. Lovesey, G. van der Laan, Circular dichroism of second harmonic generation response. *Phys. Rev. B* **100**, 245112 (2019).

36. G. van der Laan, S. W. Lovesey, Electronic multipoles in second harmonic generation and neutron scattering. *Phys. Rev. B* **103**, 125124 (2021).

37. C. Ophus, Four-dimensional scanning transmission electron microscopy (4D-STEM): From scanning nanodiffraction to ptychography and beyond. *Microsc. Microanal.* **25**, 563–582 (2019).

38. M. W. Tate, P. Purohit, D. Chamberlain, K. X. Nguyen, R. Hovden, C. S. Chang, P. Deb, E. Turgut, J. T. Heron, D. G. Schlom, D. C. Ralph, G. D. Fuchs, K. S. Shanks, H. T. Philipp, D. A. Muller, S. M. Gruner, High dynamic range pixel array detector for scanning transmission electron microscopy. *Microsc. Microanal.* **22**, 237–249 (2016).

39. K. X. Nguyen, Y. Jiang, M. C. Cao, P. Purohit, A. K. Yadav, P. García-Fernández, M. W. Tate, C. S. Chang, P. Aguado-Puente, J. Íñiguez, F. Gomez-Ortiz, S. M. Gruner, J. Junquera, L. W. Martin, R. Ramesh, D. A. Muller, Transferring orbital angular momentum to an electron beam reveals toroidal and chiral order. arXiv:2012.04134 [cond-mat.mtrl-sci] (8 December 2020).

40. B. H. Savitzky, L. Hughes, K. C. Bustillo, H. D. Deng, N. L. Jin, E. G. Lomeli, W. C. Chueh, P. Herring, A. Minor, C. Ophus, py4DSTEM: Open source software for 4D-STEM data analysis. *Microsc. Microanal.* **25**, 124–125 (2019).

41. J. C. Wojdeł, P. Hermet, M. P. Ljungberg, P. Ghosez, J. Íñiguez, First-principles model potentials for lattice-dynamical studies: General methodology and example of application to ferroic perovskite oxides. *J. Phys. Condens. Matter* **25**, 305401 (2013).

42. P. García-Fernández, J. C. Wojdeł, J. Íñiguez, J. Junquera, Second-principles method for materials simulations including electron and lattice degrees of freedom. *Phys. Rev. B* **93**, 195137 (2016).

43. G. W. Farnell, I. A. Cermak, P. Silvester, S. Wong, "Capacitance and field distributions for interdigital surface-wave transducers" (McGill University Montreal (Quebec) Department of Electrical Engineering, 1969).

44. L.-Q. Chen, Phase-field method of phase transitions/domain structures in ferroelectric thin films: A review. *J. Am. Ceram. Soc.* **91**, 1835–1844 (2008).

45. Y. L. Li, S. Y. Hu, Z. K. Liu, L. Q. Chen, Effect of electrical boundary conditions on ferroelectric domain structures in thin films. *Appl. Phys. Lett.* **81**, 427–429 (2002).

46. L. Q. Chen, J. Shen, Applications of semi-implicit Fourier-spectral method to phase field equations. *Comput. Phys. Commun.* **108**, 147–158 (1998).

47. F. Xue, J. J. Wang, G. Sheng, E. Huang, Y. Cao, H. H. Huang, P. Munroe, R. Mahjoub, Y. L. Li, V. Nagarajan, L. Q. Chen, Phase field simulations of ferroelectrics domain structures in PbZrxTi1−xO3 bilayers. *Acta Mater.* **61**, 2909–2918 (2013).

48. J. C. Wojdeł, J. Íñiguez, Ferroelectric transitions at ferroelectric domain walls found from first principles. *Phys. Rev. Lett.* **112**, 247603 (2014).

49. P. A. Franken, A. E. Hill, C. W. Peters, G. Weinreich, Generation of optical harmonics. *Phys. Rev. Lett.* **7**, 118–119 (1961).

50. S. W. Lovesey, E. Balcar, A guide to electronic multipoles in photon scattering and absorption. *J. Phys. Soc. Jpn.* **82**, 021008 (2012).

51. H. A. Kramers, W. Heisenberg, Über die streuung von strahlung durch atome. *Z. Phys.* **31**, 681–708 (1925).

52. B. R. Judd, Optical absorption intensities of rare-earth ions. *Phys. Rev.* **127**, 750–761 (1962).

53. G. S. Ofelt, Intensities of crystal spectra of rare-earth ions. *J. Chem. Phys.* **37**, 511–520 (1962).

54. B. H. Savitzky, L. A. Hughes, S. E. Zeltmann, H. G. Brown, S. Zhao, P. M. Pelz, E. S. Barnard, J. Donohue, L. R. DaCosta, T. C. Pekin, E. Kennedy, M. T. Janish, M. M. Schneider, P. Herring, C. Gopal, A. Anapolsky, P. Ercius, M. Scott, J. Ciston, A. M. Minor, C. Ophus, py4DSTEM: A software package for multimodal analysis of four-dimensional scanning transmission electron microscopy datasets. arXiv:2003.09523 [cond-mat.mtrl-sci] (20 March 2020).

55. H. A. Dürr, E. Dudzik, S. S. Dhesi, J. B. Goedkoop, G. van der Laan, M. Belakhovsky, C. Mocuta, A. Marty, Y. Samson, Chiral magnetic domain structures in ultrathin FePd films. *Science* **284**, 2166–2168 (1999).

56. M. Bode, M. Heide, K. von Bergmann, P. Ferriani, S. Heinze, G. Bihlmayer, A. Kubetzka, O. Pietzsch, S. Blügel, R. Wiesendanger, Chiral magnetic order at surfaces driven by inversion asymmetry. *Nature* **447**, 190–193 (2007).

57. P. Schoenherr, J. Müller, L. Köhler, A. Rosch, N. Kanazawa, Y. Tokura, M. Garst, D. Meier, Topological domain walls in helimagnets. *Nat. Phys.* **14**, 465–468 (2018).

58. R. Streubel, C.-H. Lambert, N. Kent, P. Ercius, A. T. N'Diaye, C. Ophus, S. Salahuddin, P. Fischer, Experimental evidence of chiral ferrimagnetism in amorphous GdCo films. *Adv. Mater.* **30**, 1800199 (2018).

59. E. H. Chen, O. Gaathon, M. E. Trusheim, D. Englund, Wide-field multispectral super-resolution imaging using spin-dependent fluorescence in nanodiamonds. *Nano Lett.* **13**, 2073–2077 (2013).

60. G. Chen, T. Ma, A. T. N'Diaye, H. Kwon, C. Won, Y. Wu, A. K. Schmid, Tailoring the chirality of magnetic domain walls by interface engineering. *Nat. Commun.* **4**, 2671 (2013).

61. G. Catalan, J. Seidel, R. Ramesh, J. F. Scott, Domain wall nanoelectronics. *Rev. Mod. Phys.* **84**, 119–156 (2012).

62. V. Stepkova, P. Marton, J. Hlinka, Stress-induced phase transition in ferroelectric domain walls of $BaTiO_3$. *J. Phys. Condens. Matter* **24**, 212201 (2012).

63. Y.-J. Wang, Y.-L. Zhu, X.-L. Ma, Chiral phase transition at 180° domain walls in ferroelectric $PbTiO_3$ driven by epitaxial compressive strains. *J. Appl. Phys.* **122**, 134104 (2017).

64. N. D. Mermin, The homotopy groups of condensed matter physics. *J. Math. Phys.* **19**, 1457–1462 (1978).

65. Z. Hong, A. R. Damodaran, F. Xue, S.-L. Hsu, J. Britson, A. K. Yadav, C. T. Nelson, J.-J. Wang, J. F. Scott, L. W. Martin, R. Ramesh, L.-Q. Chen, Stability of polar vortex lattice in ferroelectric superlattices. *Nano Lett.* **17**, 2246–2252 (2017).
